# Antifungal mechanism of cell-free supernatant produced by *Trichoderma virens* and its efficacy for the control of pear Valsa canker

**DOI:** 10.3389/fmicb.2024.1377683

**Published:** 2024-04-17

**Authors:** Yang Zhang, Ying Lu, Zhaoyang Jin, Bo Li, Li Wu, Yujian He

**Affiliations:** ^1^School of Chemical Sciences, University of Chinese Academy of Sciences, Beijing, China; ^2^School of Future Technology, University of Chinese Academy of Sciences, Beijing, China; ^3^Institute of Farmland Water Conservancy and Soil Fertilizer, Xinjiang Academy of Agricultural and Reclamation Science, Shihezi City, China

**Keywords:** *Valsa pyri*, *Trichoderma virens*, cell-free supernatant, antifungal activity, reactive oxygen species

## Abstract

**Introduction:**

Pear Valsa canker, caused by *Valsa pyri* (*V. pyri*), poses a major threat to pear production. We aimed to assess the effectiveness of the cell-free supernatant (CFS) produced by *Trichoderma virens* (*T. virens*) to control the development of pear Valsa canker and reveal the inhibitory mechanism against the pathogenic fungi.

**Results:**

Using morphological characteristics and phylogenetic analysis, the pathogen G1H was identified as *V. pyri*, and the biocontrol fungus WJ561 was identified as *Trichoderma virens*. CFS derived from WJ561 exhibited strong inhibition of mycelial growth and was capable of reducing the pathogenicity of *V. pyri* on pear leaves and twigs. Scanning electron microscopy (SEM) observations revealed deformations and shrinkages in the fungal hyphae treated with CFS. The CFS also destroyed the hyphal membranes leading to the leakage of cellular contents and an increase in the malondialdehyde (MDA) content. Additionally, CFS significantly inhibited the activities of catalase (CAT) and superoxide dismutase (SOD), and downregulated the expression of antioxidant defense-related genes in *V. pyri*, causing the accumulation of reactive oxygen species (ROS). Artesunate, identified as the main component in CFS by liquid chromatograph-mass spectrometry (LC–MS), exhibited antifungal activity against *V. pyri*.

**Conclusion:**

Our findings demonstrate the promising potential of *T. virens* and its CFS in controlling pear Valsa canker. The primary inhibitory mechanism of CFS involves multiple processes, including membrane damage and negatively affecting enzymatic detoxification pathways, consequently leading to hyphal oxidative damage of *V. pyri*. This study lays a theoretical foundation for the utilization of *T. virens* to control *V. pyri* in practical production.

## 1 Introduction

The *Pyrus*, commonly known as the pear, is a globally cultivated fruit that holds a significant position in the fruit industry, following only the consumption of oranges and apples. Its widespread popularity can be attributed to its high nutritional value, which includes dietary fiber, vitamin C, potassium, and other phytonutrients that provide various health benefits. Plant canker disease, caused by pathogenic bacteria or fungi, is highly prevalent and detrimental to plant productivity, bringing significant economic implications for growers and the industry, and can also impact consumers (Song et al., 2020; Naqvi et al., 2022). *V. pyri*, responsible for pear Valsa canker, is considered one of the most severe pear tree diseases and is prevalent in several regions (Song et al., 2020). Similar to what *Valsa mali* (*V. mali*) does to apple trees, this pathogen can also penetrate the phloem and trigger cankers on the pear branches and trunks, which affects the growth of the trees and fruit quality and may even lead to the death of the tree (Yin et al., 2015). The cankers may also serve as entry points for other pathogens, leading to further damage.

Due to the economic importance of pears, it is crucial to take preventive measures to control the spread of pear Valsa canker. Over the past decades, conventional methods for managing this disease have included physical and chemical interventions. However, physical techniques, such as removing diseased lesions and pruning infected branches, have proven insufficient due to their inability to eradicate the disease effectively. In some cases, they may even exacerbate tree harm (Chen et al., 2016; He et al., 2018). While chemical fungicides, including copper-based compounds, have been utilized for the control of pear Valsa canker, the penetration of the pathogen into the deep xylem of the host renders most chemical treatments ineffective in the inner portions of the tree trunk, thereby allowing unimpeded pathogen progression (Abe et al., 2007; Song et al., 2020). In addition, escalating concerns about potential toxicity and ecological risks posed by fungicide residues have catalyzed the pursuit of preferable alternative methods to address the disease (Zubrod et al., 2019; Ismaila et al., 2023). For sustainable agricultural practices, innovative strategies such as biological control have surfaced as promising tools for controlling pear Valsa canker and reducing the adverse effects of traditional methods.

The utilization of biocontrol bacteria in controlling pear Valsa canker induced by *Valsa pyri* is of utmost importance. An excellent example of such biocontrol bacteria is *Bacillus subtilis*, which can be employed to combat pear Valsa canker, effectively controlling the spread of the disease (Song et al., 2020). Moreover, the *Saccharothrix yanglingensis* strain Hhs.015 has demonstrated comparable efficacy in controlling Valsa canker on apple trees (Li et al., 2016). Given the susceptibility of biocontrol agents to environmental nuances, the inherent adaptability and ubiquity of *Trichoderma* species have rendered them extensively viable (Zhang X. et al., 2020; Zhu et al., 2022). Consequently, their application has been widely embraced in addressing diverse plant pathogens (Zhang et al., 2016; Wu et al., 2017). Mycoparasitism, antibiosis, competition, and systemic acquired resistance (SAR) constitute the principal modes through which *Trichoderma* exerts its biocontrol activities (Dutta et al., 2023). The antibiosis mechanism involved in the interaction between *Trichoderma* and pathogen is driven by a variety of primary and secondary metabolites secreted by *Trichoderma*. These metabolites, including fatty acids, organic acids, terpenes, amides, and others (Zhang et al., 2018; Gan et al., 2022; Zaid et al., 2022), have broad-spectrum antimicrobial activity in their ecological niches, can destroy cell membrane, and inhibit mycelium growth or spore germination of the targeted pathogen. A previous study showed that fermentation of *Trichoderma longibrachiatum* T6 could exert antifungal effects on the pathogen *V. mali* of apple *Valsa* canker, such as inhibiting conidia germination, altering mycelial morphology, and blocking growth (Zhu et al., 2022). Research involving an assay targeting *Rhizoctonia solani* and nematode mortality has demonstrated that the suppression of these pathogens can be attributed to the antibiosis mechanism of *Trichoderma virens* metabolites (Halifu et al., 2020; Moo-Koh et al., 2022). These metabolites functioned as inhibitors, facilitating the penetration of *Trichoderma* hyphae into prey fungi and hampering vital processes such as cell wall synthesis, growth, reproduction, sporulation, nutrient uptake, metabolite production in target pathogens, and induction of defense system (Halifu et al., 2020; Gan et al., 2022; Zhu et al., 2022). However, the function of *T. virens* on antifungal activity on *V. pyri* remains to be explored.

The purpose of this study was to reveal the effect and mechanism of *T. virens* on pear Valsa canker and to provide a theoretical basis for using *T. virens* to control this disease.

## 2 Materials and methods

### 2.1 Fungal isolation and identification

#### 2.1.1 Pathogen and *Trichoderma* isolation

In the year 2021, we collected eight bark samples from two Hongxiao’ pear (*Pyrus* sp.) trees, which displayed symptoms of pear Valsa canker in Huaibei Town, Beijing City, China. The pathogenic fungi were isolated from the infected bark using the tissue isolation method. This involved carefully excising small pieces (0.5 cm) from the edges of the diseased bark lesions using a sterile scalpel. The excised tissues were then subjected to surface sterilization by treating them with tap water, followed by immersion in 75% ethanol for 2 min. Subsequently, it underwent three washes with sterile distilled water before being positioned on sterilized potato dextrose agar (PDA) plates at 25°C for 3 to 5 days. Regular inspections were carried out to monitor any fungal growth. Colonies developed from the infected tissues were transferred to new PDA plates using the hyphal tip culture method to obtain pure cultures. The purified isolate, named G1H, was further subcultured on PDA for 3 days and kept on PDA slants at 4°C, and spores were frozen in 15% sterile glycerin at −80°C. Then, the classification position was confirmed by morphological observation and sequencing. Endophytic *Trichoderma* was isolated from ginger rhizomes in Wuji County, Shijiazhuang City, Hebei Province, China. The fungal isolation process was conducted as previously described. The rhizomes were incubated on PDA for 5 to 7 days at 28°C to obtain purified *Trichoderma* spp., among which an isolate *T. virens*, named WJ561, was discovered by morphological observation. The phylogenetic analysis was confirmed by DNA sequencing.

#### 2.1.2 DNA extraction, sequencing, and phylogenetic analysis

Isolates were grown on PDA for 3 to 5 days under the conditions described earlier. Approximately 100 mg of mycelium was gathered from the colonies formed in potato dextrose broth (PDB) medium and placed in sterile tubes. A Sangon Biotech Genome Extraction Kit (Shanghai Sangon Biotech Co., Ltd., China) was used to extract DNA, according to the manufacturer’s instructions. For PCR and sequencing analysis, the region of nuclear rDNA containing the internal transcribed spacer regions 1 and 2 and the 5.8S rDNA gene region (ITS) was amplified using the ITS1 and ITS4 primers (Innis et al., 1990). In addition, G1H was subjected to analyses of two additional loci, the translation elongation factor-1 alpha (EF1α) gene and the β-tubulin (Btu) gene (Wang et al., 2014). The partial sequence of the EF1α gene was amplified with primers EF1-728F and EF1-986R (Carbone and Kohn, 1999). A partial Btu gene sequence was amplified using primers Bt2a and Bt2b (Glass and Donaldson, 1995). In addition, for the identification of *Trichoderma* (Li et al., 2023), a fragment of the gene translation elongation factor 1-α (TEF1-α) was amplified using EF1-728F and TEF1LLErev primers (Chaverri et al., 2003; Jaklitsch et al., 2005), and RNA polymerase II subunit B (RPB2) gene was amplified using fRPB2-5f and fRPB2-7cr (Liu et al., 1999) primers. PCR conditions are presented in Table 1. The 25 μL PCR reaction system comprising 12.5 μL of 2× PCR MasterMix (Tiangen, Beijing, China), 1 μL of each primer, 1 μl of template DNA, and 9.5 μL of ddH_2_O. The successful amplification was verified through gel electrophoresis.

**Table 1:** PCR primers and conditions used for DNA amplification.

Genus	Locus	Prime	Sequence (5′ → 3′)	PCR conditions	References
*Valsa*/ *Trichoderma*	ITS	ITS1	TCCGTAGGTGAACCTGCGG	4 min at 94°C; 30 cycles of 45 s at 94°C, 45 s at 55°C and 1 min at 72°C; and 10 min at 72°C.	Innis et al. (1990)
		ITS4	TCCTCCGCTTATTGATATGC		
*Valsa*	EF1α	EF1-728F	CATCGAGAAGTTCGAGAAGG	4 min at 94°C; 38 cycles of 30 s at 94°C, 30 s at 55°C and 30 s at 72°C; and 5 min at 72°C.	Carbone and Kohn (1999)
		EF1-986R	TACTTGAAGGAACCCTTACC		
	Btu	Bt2a	GGTAACCAAATCGGTGCTGCTTTC	4 min at 94°C; 38 cycles of 30 s at 94°C, 30 s at 61°C and 30 s at 72°C; and 5 min at 72°C.	Glass and Donaldson (1995)
		Bt2b	GGTAACCAAATCGGTGCTGCTTTC		
*Trichoderma*	TEF1-α	EF1-728F	CATCGAGAAGTTCGAGAAGG	5 min at 95°C; 30 cycles of 1 min at 95°C, 90 s at 55°C and 90 s at 72°C; and 10 min at 72°C.	Chaverri et al. (2003) and Jaklitsch et al. (2005)
		TEF1LLErev	AACTTGCAGGCAATGTGG		
	RPB2	fRPB2-5f	GAYGAYMGWGATCAYTTYGG	2 min at 95°C; 36 cycles of 1 min at 95°C, 1 min at 52°C to each second with increment of 0.2°C and 1 min at 72°C; and 7 min at 72°C.	Liu et al. (1999)
		fRPB2-7cr	CCCATRGCTTGYTTRCCCAT		

Amplification products were purified and sequenced bidirectionally at Shanghai Sangon Biotech Co., Ltd. Basic Local Alignment Search Tool (BLAST)^1^ analysis was conducted for each gene locus to identify the isolates. The sequences were submitted to GenBank. For phylogenetic analysis, we retrieved homologous ITS, TEF1-α (or EF1α), Btu, and RPB2 gene sequences from GenBank, which were aligned using BioEdit software (version 7.0.9). The PhyloSuite v1.2.3 (Zhang D. et al., 2020; Xiang et al., 2023) was used to concatenate the gene sequences. ModelFinder v2.2.0 (Kalyaanamoorthy et al., 2017) was used to select the best-fit partition model (Edge-linked) using the BIC criterion, and the maximum likelihood (ML) tree was performed using IQ-TREE v (Nguyen et al., 2015) with 1,000 standard bootstraps in PhyloSuite v1.2.3. The ML trees were visualized using FigTree v1.4.4, with bootstrap values (≥ 70%) being given near nodes.

### 2.2 *In vitro* antagonism experimental procedures

#### 2.2.1 Dual culture assay

Dual confrontations between *T. virens* WJ561 and *V. pyri* G1H were carried out as previously described (Wonglom et al., 2019). Unless otherwise indicated, the WJ561 strains were grown at 28°C, and the G1H strains were cultivated at 25°C. In brief, an agar plug of 6 mm diameter colonized by 5-day-old WJ561 culture was placed 5 cm from the border on the opposite side of the plate on which a mycelial plug inhabited by G1H was grown. Because WJ561 grows much more slowly than G1H, we initiated WJ561 culture 1 day before co-cultivation. Sterile PDA plugs maintained alone were used as controls. Dual cultures were performed three times with 15 replicates, and the radii of the colonies were measured after 3 days of incubation, to calculate the percentage of inhibition. The growth inhibition of fungal hyphae is calculated as follows:


$$\mathrm{Inhibition rate}\;\left(\%\right)=\left(1-\frac{\text{Rt}}{\text{Rc}}\right)\times 100\%$$

Rt is the radius (cm) of the treatment group and Rc is the radius (cm) of the control group.

#### 2.2.2 Volatile organic compounds (VOCs) antifungal bioassay

The antagonistic activity of volatile organic compounds from *T. virens* WJ561 against the *V. pyri* G1H was assessed using the sandwiched Petri plate assays (Li et al., 2018; Halifu et al., 2020). After inoculating WJ561 and G1H on the PDA plate, a plate containing G1H agar plug was placed on top of the WJ561 plate, sealed with two layers of Parafilm, and incubated at 25°C. This configuration ensured that there was no direct interaction between the two fungi. Each plate of G1H was also sandwiched with an un-inoculated PDA plate as a control treatment. Colony diameter of G1H was measured 3 days later. We evaluated the inhibitory effect of WJ561 VOCs on G1H in the same way, except that 1-day-old and 2-day-old WJ561 culture plates were used to ensure enough VOCs. Each treatment included 15 biological replicates and was conducted three times. The diameter of the colony was measured to evaluate the inhibition of the growth of G1H as previously described.

#### 2.2.3 Antifungal potency of *Trichoderma virens* WJ561 cell-free supernatant

To evaluate the antifungal efficacy of cell-free supernatant produced by *T. virens* WJ561, eight WJ561 mycelial plugs were introduced into 100 mL of PDB liquid medium and incubated at 28°C for 2, 4, 6, 10, and 14 days with shaking at 150 rpm. The CFS was first collected into tubes using 12 layers of lens wiping paper and re-filtered twice using a 0.1-μm sterile syringe filter (Guangzhou Jet Bio-Filtration Co., Ltd.). In total, 10 mL of sterile PDA was mixed with 5 mL of CFS. The same amount of PDB liquid medium instead of the CFS was used as the control. A mycelial plug of G1H was placed at the center of the PAD plate and incubated at 25°C for 2 days. The potency of CFS was determined by calculating the mycelial growth rate (Li et al., 2020). The experiment was performed with three replications and repeated three times. Furthermore, a mycelial plug of G1H was incubated in PDB with varying working volume fractions (0, 5, 10, and 15%) of WJ561 CFS while shaking at 25°C and 180 rpm for 72 h. Then, the weight of the mycelia was measured after collecting and drying.

### 2.3 Detached twig and leaf assays

The effectiveness of *T. virens* WJ561 was tested on detached twigs and leaves in the laboratory. Healthy twigs and leaves from ‘Hongxiao’ pear were treated as previously described (Zang et al., 2007; Zhao et al., 2022). The twigs were prepared by cutting them into 12 cm segments, washing them with tap water, and disinfecting them with 75% alcohol. After rinsing them with sterile water, both ends were sealed with paraffin wax once dry. All twigs were treated with hot iron nail caps (5 mm in diameter) in the neutral position to scald them. On each segment, CFS with different working volume fractions (0, 5, 10, and 15%) was uniformly applied three times and left to air dry for 15 min. Following this, a 6-mm diameter mycelial disk of G1H was inoculated on the scalded area of each twig. These inoculated twigs were placed in a clean container at 25°C and covered with fresh-keeping film. The humidity was regulated using a sterile filter paper. The lesion size was recorded after 5 days and repeated in triplicate.

After surface disinfection, the leaves were repeatedly wiped with 500 μL of WJ561 CFS at concentrations of 0, 5, 10, and 15%. The treated leaves were naturally air-dried and then punctured with sterile stainless needles to form a symmetrically uniform hole (1 mm wide). Then, a G1H plug was inoculated in the pretreated holes. The leaves were then placed on sterilized filter paper to maintain humidity and then incubated at 25°C. Photos of the lesions were recorded after 4 days, and the experiments were repeated twice with three biological replicates.

### 2.4 Stability of *Trichoderma virens* WJ561 cell-free supernatant *Valsa pyri*

The stability of CFS was evaluated as described (Ghribi et al., 2012; Zhang et al., 2016; Li et al., 2020). Thermal tolerance was tested by subjecting WJ561 CFS to temperatures of 4, 50, 80, 100, and 121°C for 30 min and then measuring the remaining antifungal activity after cooling to room temperature. Acid–base stability was assessed by adjusting the pH of the filtrates to 1, 3, 5, 7, 9, 11, and 13 with 0.1 M HCl or NaOH for 2 h. Then, the filtrates were readjusted to the original pH values before determining their antifungal activity. Radiation stability was examined by exposing the filtrates to ultraviolet light for 0.5, 1, 2, 3, and 4 h, respectively. To determine the effect of storage time on CFS stability, antifungal assays were performed after the filtrates had been stored at −20°C for 4, 8, and 12 months. Finally, the impact of different CFS treatments on the mycelial growth of G1H on the PDA plate was determined according to step 2.2.3. All experiments were repeated in triplicate, and each treatment contained three plates under the same conditions.

### 2.5 Effect of *Trichoderma virens* WJ561 cell-free supernatant on mycelial morphology of *Valsa Pyri* G1H

Fresh mycelia of G1H were treated with 0, 5, 10%, or 15% WJ561 CFS at 25°C for 24 h. The treated mycelia were filtered and fixed with 2.5% glutaraldehyde for 24 h. This was followed by dehydration with graded ethanol (30, 50, 70, and 90%, v/v) as described (Fan et al., 2023). The dried samples were coated with gold and subsequently observed using scanning electron microscopy (SEM, Hitachi Regulus 8,220, Japan).

### 2.6 Determination of the integrity of cell membranes

A few treated mycelia obtained in step 2.5 were immersed in 5 mM propidium iodide (PI, Shanghai Acmec Biochemical Technology Co., Ltd., Shanghai, China) for 20 min at 30°C and washed three times with a phosphate-buffered saline (PBS) (0.05 M, pH 7.2–7.4) to remove the dye. The red fluorescence was examined using laser confocal microscopy (Laica STELLARIS 5, Germany) (Zhao et al., 2022). Each group was treated with three samples, and the experiment was repeated three times.

### 2.7 Determination of malondialdehyde content and the leakage of cellular content

Mycelium samples treated in step 2.5 were homogenized in ice-cold 0.05 M PBS (pH 7.2–7.4) within an ice bath. The MDA content was determined using a commercial thiobarbituric acid (TBA) kit (Bioroyee (Beijing) Biotechnology Co., Ltd., Beijing, China). The filtrated solutions were used for the determination of nucleic acid and soluble protein exudation via the NanoDrop One (Thermo Fisher Scientific, Waltham, MA, United States), as described previously with some modifications (Cai et al., 2015; Fan et al., 2023). Each treatment consisted of three biological replicates, and the experiments were performed three times.

### 2.8 Detection of reactive oxygen species accumulation

Fresh mycelium samples treated in step 2.5 were resuspended in 0.05 M PBS and stained with 1 μM 2,7-dichlorodihydrofluorescein diacetate (H_2_DCFDA, Beijing Solarbio Science & Technology Co., Ltd., Beijing, China) for 20 min at 37°C and then rinsed three times with PBS to remove residual dye. Confocal laser microscopy was used for fluorescence observation as descri bed by Kai et al., 2020.

### 2.9 Enzyme activity assay and real-time fluorescence quantitative polymerase chain reaction assay

The enzyme activities of catalase and superoxide dismutase were determined using commercial kits (Beijing Solarbio Science & Technology Co., Ltd., Beijing, China). The total RNA of *V. pyri* G1H was extracted from the mycelia collected in step 2.5 using a commercial kit (Vazyme, Nanjing, China), and the cDNA was achieved through a RevertAid First Strand cDNA Synthesis Kit (Thermo Fisher Scientific, Waltham, MA, United States). RT-qPCR was performed in a Real-Time PCR System (Applied Biosystems). The PCR reaction conditions were as follows: one cycle at 50°C for 2 min and 95°C for 2 min, then 40 cycles of 15 s at 95°C and 1 min at 60°C. The gene expression level was normalized by the internal reference gene *actin* with the 2^−∆∆Ct^ method (Arocho et al., 2006; He et al., 2018). Specific primers for gene evaluation are shown in Table 2. Each group consisted of three biological replicates, and the experiments were repeated twice.

**Table 2:** Primers used for the RT-qPCR analysis.

Gene	Accession Number	Primer Name	Primer Sequences (5′ → 3′)
*Cat*	KN714681.1	CAT-F1	ACCCTCTTGTCTACGAGAACCTT
		CAT-R1	GGCGACGGAATCATAACCA
*Sod*	KN714874.1	SOD-F1	TGACCGCACCGACGAGAC
		SOD-R1	GGAGTGGGGTCCAATCAGC
*Actin*	KN714822.1	Actin-F1	CCGAGGCTCCTATCAACCC
		Actin-R1	GTAGATGGGGACAACGTGAGTG

### 2.10 Untargeted metabolic analysis of *Trichoderma virens* WJ561

To explore the types and abundance of metabolites produced by WJ561, six biological replicates of 4-day-old WJ561 CFS were collected and frozen in liquid nitrogen until used for untargeted metabolomics analysis. In total, 1 mL of the freeze-dried samples was resuspended in 100 μL of pre-chilled 80% methanol by vortexing and incubated on ice for 5 min. The resulting samples were centrifuged at 15,000 g at 4°C for 15 min. The obtained supernatant was diluted with LC–MS grade water to a final concentration containing 53% methanol. The samples were then transferred to a new tube and centrifuged at 15,000 *g* at 4°C for 15 min. Finally, the supernatant was injected into the LC–MS/MS system for analysis.

LC–MS analyses were performed on a Vanquish UHPLC system (Thermo Fisher Scientific, Waltham, MA, United States), coupled to an Orbitrap A Q Exactive^TM^ HF mass spectrometer (Thermo Fisher Scientific, Waltham, MA, United States), in Novogene Co., Ltd. (Beijing, China). Samples were injected onto a HypersilGoldcolumn (100 × 2.1 mm, 1.9 μm) using a 12-min linear gradient at a flow rate of 0.2 mL/min. The eluents for the positive polarity mode were eluent A (0.1% formic acid in water) and eluent B (methanol). The eluents for the negative polarity mode were eluent A (5 mM ammonium acetate, pH 9.0) and eluent B (methanol). The gradient program of eluent B was set as follows: 0–1.5 min for 2%; 1.5–3 min for 2 to 85%; 3–10 min for 85 to 100%; 10–10.1 min for 100 to 2%; 10.1–11 for 2%; 11–12 min for 2%. A Q Exactive^TM^ HF mass spectrometer was operated in positive/negative polarity mode with a spray voltage of 3.5 kV, capillary temperature of 320°C, sheath gas flow rate of 35 psi, and aux gas flow rate of 10 L/min, S-lens RF level of 60, and aux gas heater temperature of 350°C.

The raw data were processed using Compound Discoverer 3.3 software (CD 3.3, Thermo Fisher Scientific, Waltham, MA, United States) for baseline filtering, peak alignment, picking, and normalization to produce peak intensities for retention time (RT) and m/z data pairs. The mzCloud,^2^ mzVaultand, and MassList databases were used to identify metabolites. These metabolites were annotated using the HMDB database. Statistical analyses were performed using statistical software R (version 3.4.3), Python (version 2.7.6), and CentOS (CentOS release 6.6).

### 2.11 Inhibitory artesunate against *Valsa pyri*

Antifungal tests were conducted to evaluate the effects on the mycelial growth of *V. pyri*, as previously described (Wang et al., 2022). To determine the contact effects, artesunate was dispersed in dimethyl sulfoxide (DMSO) at a final concentration of 1% and added to tepid PDA immediately before emptying the Petri dishes. Concentrations of artesunate ranging from 0.03 to 3 mg/mL were tested. The controls received the same quantity of 1% DMSO mixed with PDA. After solidification, a disk cut from the actively growing front of a 3-day-old colony of the desired pathogenic fungi *V. pyri* G1H was aseptically placed in the center of each treatment plate. Following the same steps, we evaluated the control efficacy of 3 mg/mL lauric acid, arachidonic acid, and linoleic acid against *V*. *pyri*. All Petri dishes were then incubated at 25°C and observed after 2 days, to calculate the growth inhibition of fungal hyphae. The experiment was performed in triplicate with three biological replicates.

### 2.12 Statistical analysis

The statistical analyses were conducted using IBM SPSS 26.0 and Excel 2016. Normality (Shapiro–Wilk test, *p* > 0.05) and variance homogeneity (Levene’s test, *p* > 0.05) were checked, and parametric tests were used. Data results were analyzed using a one-way analysis of variance (ANOVA), Welch’s test, or Kruskal–Wallis H test and presented as the mean ± standard error of the least squares means. According to Duncan’s multiple range test, Welch’s test, and the Kruskal–Wallis test, a *p*-value of <0.05 was accepted as statistically significant. Different letters indicated the significant difference among the treatments.

## 3 Results

### 3.1 Pathogen isolation and identification

**Figure 1:**
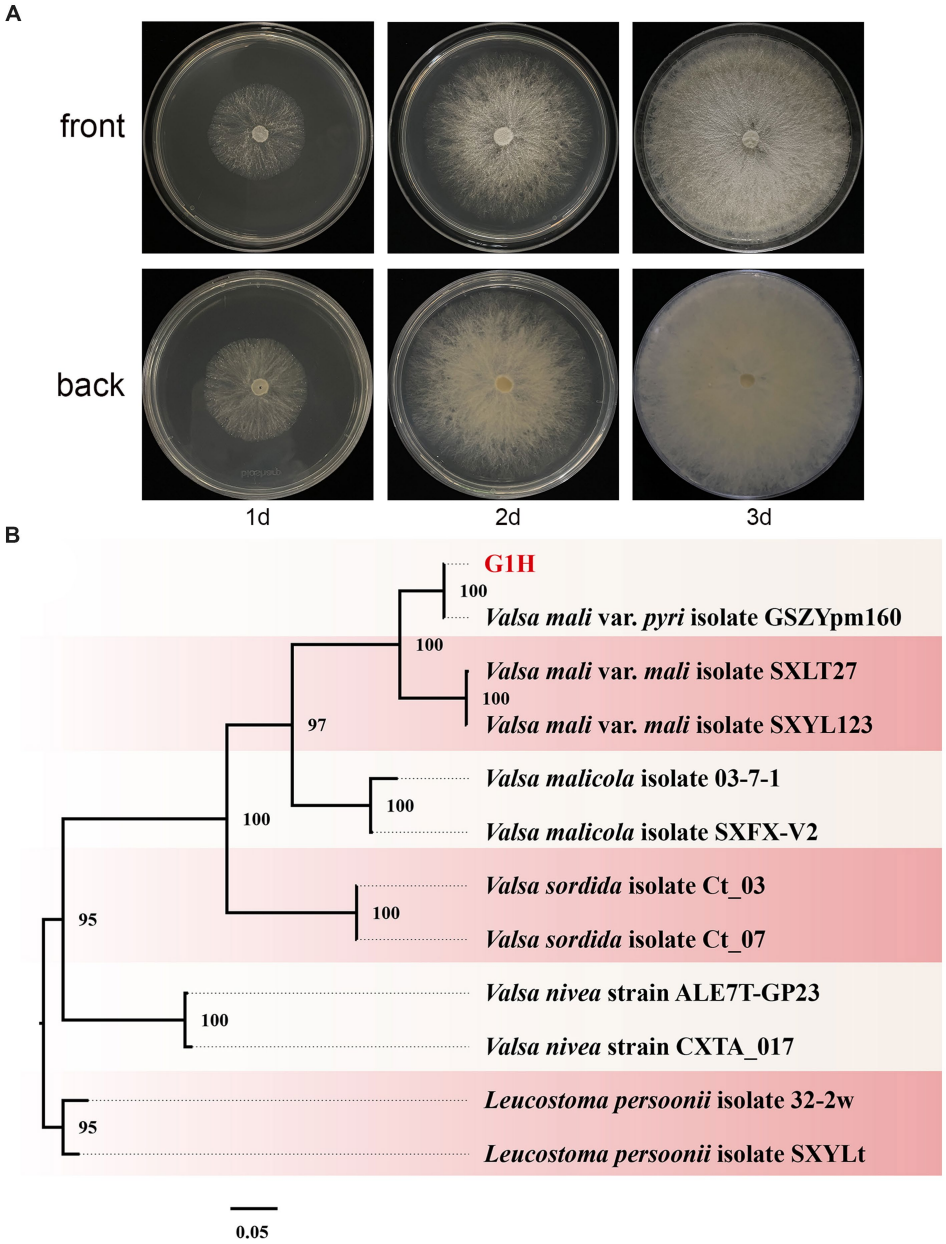
Colony appearance and maximum likelihood phylogenetic tree of *Valsa pyri* G1H. **(A)** Colony appearance of G1H at 1, 2, and 3 days of incubation from above and below. **(B)** Phylogenetic tree of G1H based on the combined ITS, EF1α, and Btu genes. Bootstrap support values equal to or greater than 70% are shown at the nodes. *Leucostoma persoonii* isolate SXYLt and *Leucostoma persoonii* isolate 32-2w were used as the outgroup. G1H was highlighted in red.

Pear Valsa canker is a severe disease that can cause significant damage to tree productivity. The symptoms are visually distinct and include cankers on pear trunks, scaffold limbs, and diebacks of twigs. To investigate the pathogens responsible for pear Valsa canker, we isolated fungi from the infected pear bark. Among the isolates, morphological observations revealed that G1H can proliferate normally on PDA medium and form colonies (Figure 1A). The radial growth rate of mycelia was rapid, completely covering the plate within no more than 3 days. Colonies were milk-white, thin, with a dense surface. The medium on the back of the plate was milk-yellow.

The ITS, EF1α, and Btu gene sequences of G1H were used as a query in a BLAST search of the NCBI GenBank database. Therefore, as a next step, a phylogenetic analysis was conducted using a multilocus dataset of concatenated ITS, EF1α, and Btu obtained from 12 samples. *Leucostoma persoonii* isolate SXYLt and *Leucostoma persoonii* isolate 32-2w were used as the outgroup. For more detailed information on the specific characteristics of each isolate, please refer to Table 3. The analysis revealed that G1H was in the same clade with *Valsa mali* var. *pyri* isolate GSZYpm160, and the clade classification was strongly supported by bootstrap analysis (Figure 1B). Based on the morphology and phylogeny, the pathogenic fungi could be classified into *Valsa pyri*.

**Table 3:** Strains and NCBI GenBank accession numbers used for phylogenetic analyses of G1H.

Species	Strain	Country	GenBank accession numbers		
			ITS	EF1α	Btu
*V. pyri*	G1H	China	OR122731	OR876853	OR876852
*V. pyri*	GSZYpm160	China	JN662370	JQ900328	JQ900365
*V. mali*	SXLT27	China	JN412608	JQ900322	JQ900356
*V. mali*	SXYL123	China	JN792573	JQ900311	JQ900355
*V. malicola*	03–7-1	China	JN792575	JQ900337	JQ900370
*V. malicola*	SXFX-V2	China	GU174579	JQ900335	JQ900368
*V. sordida*	Ct_03	Turkey	OR166866	OR183584	OR183586
*V. sordida*	Ct_07	Turkey	OR166867	OR183585	OR183587
*V. nivea*	ALE7T-GP23	China	OK442666	OK510872	OK510870
*V. nivea*	CXTA_017	China	ON843984	ON866836	ON866757
*L. persoonii*	SXYLt	China	JN792579	JQ900339	JQ900373
*L. persoonii*	32-2w	China	JN584644	JQ900340	JQ900374

### 3.2 *Trichoderma* isolation and identification

**Figure 2:**
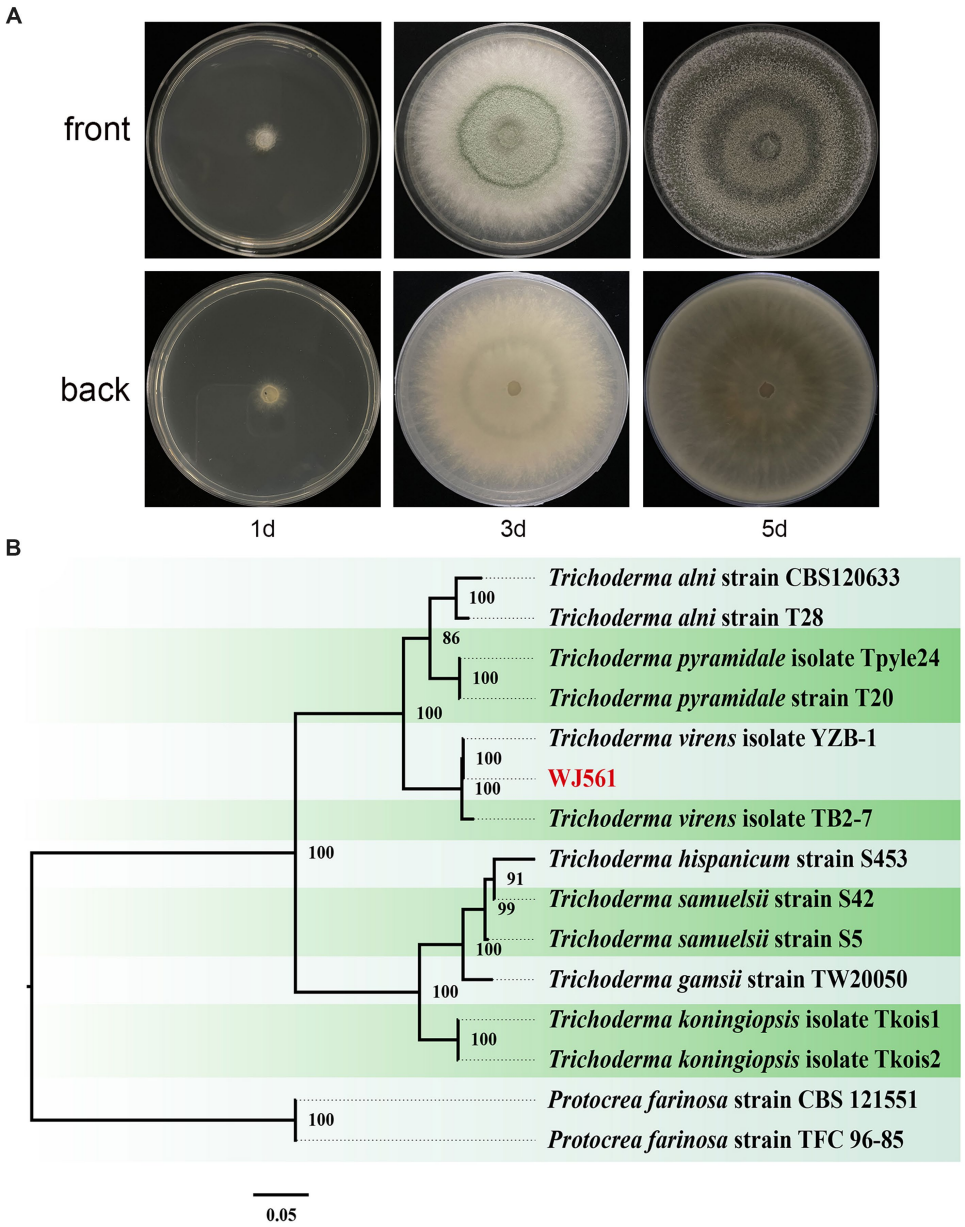
Colony appearance and maximum likelihood phylogenetic tree of *Trichoderma virens* WJ561. **(A)** Colony appearance of WJ561 at 1, 3, and 5 days of incubation from above and below. **(B)** Phylogenetic tree of WJ561 based on the combined ITS, TEF1-α, and RPB2 genes. Bootstrap support values equal to or greater than 70% are shown at the nodes. The tree was rooted in two isolates of *Protocrea farinosa* strain CBS 121551 and *Protocrea farinosa* strain TFC 96–85. WJ561 was highlighted in red.

We meticulously examined the colony shape to identify WJ561 (Figures 2A,B). After 1 day of cultivation at 28°C, all strains displayed white villous colonies on the PDA medium. We conducted a thorough analysis of the morphology of the colony to distinguish WJ561 (as shown in Figure 2A). Following 24 h of incubation at 28°C, all the strains exhibited white, hairy colonies on the PDA medium. The back of the colony is yellowish-white. By the 3rd day, light green to dull green sporulation bands emerged in the center of all media, gradually extending toward the surroundings. Colonies were dense and floccose in texture, with a fluffy surface. By the 5th day, green spores were dispersed throughout the entire plate with concentric rings, exhibiting a deep green, grayish green, or chartreuse color.

The phylogenetic trees were constructed using a dataset consisting of 15 sequences derived from 3 gene loci (ITS, TEF1-α, and RPB2) obtained from 15 isolates and 45 related sequences of *Trichoderma* and *Protocrea* species (Table 4). In the maximum likelihood phylogenetic tree, WJ561 was clustered in the same clade of *Trichoderma virens* isolate YZB-1 and *Trichoderma virens* isolate TB2-7 with 100% bootstrap support (Figure 2B). Based on the morphology and phylogeny, we identified WJ561 as *Trichoderma virens* and deposited it at the China General Microbiological Culture Collection Center (CGMCC) as CGMCC No. 40848.

**Table 4:** Strains and NCBI GenBank accession numbers used for phylogenetic analyses of WJ561.

Species	Strain	Country	GenBank accession numbers		
			ITS	TEF1-α	RPB2
*T. alni*	CBS120633	Austria	EU518651	EU498312	EU498349
*T. alni*	T28	China	KX632519	KX632633	KX632576
*T. pyramidale*	Tpyle24	China	MT102399	MT081438	MT118255
*T. pyramidale*	T20	China	KX632513	KX632627	KX632570
*T. virens*	YZB-1	China	MZ220425	OL770280	OL770281
***T. virens***	**WJ561**	**China**	**OR396899**	**OR915854**	**OR915855**
*T. virens*	TB2-7	China	MW325929	MW325734	MW325756
*T. hispanicum*	S453	Austria	JN715595	JN715659	JN715600
*T. samuelsii*	S42	Austria	JN715593	JN715652	JN715598
*T. samuelsii*	S5	Austria	JN715596	JN715651	JN715599
*T. gamsii*	TW20050	China	KU523894	KU523895	KU523896
*T. koningiopsis*	Tkois1	China	MT102394	MT081435	MT081443
*T. koningiopsis*	Tkois2	China	MT102395	MT081436	MT118250
*P. farinosa*	CBS 121551	Austria	EU703910	EU703889	EU703935
*P. farinosa*	TFC 96–85	Austria	EU703920	EU703895	EU703937

### 3.3 Inhibition of *Valsa pyri* growth in PDA By *Trichoderma virens WJ561*

#### 3.3.1 Dual culture assay

**Figure 3:**
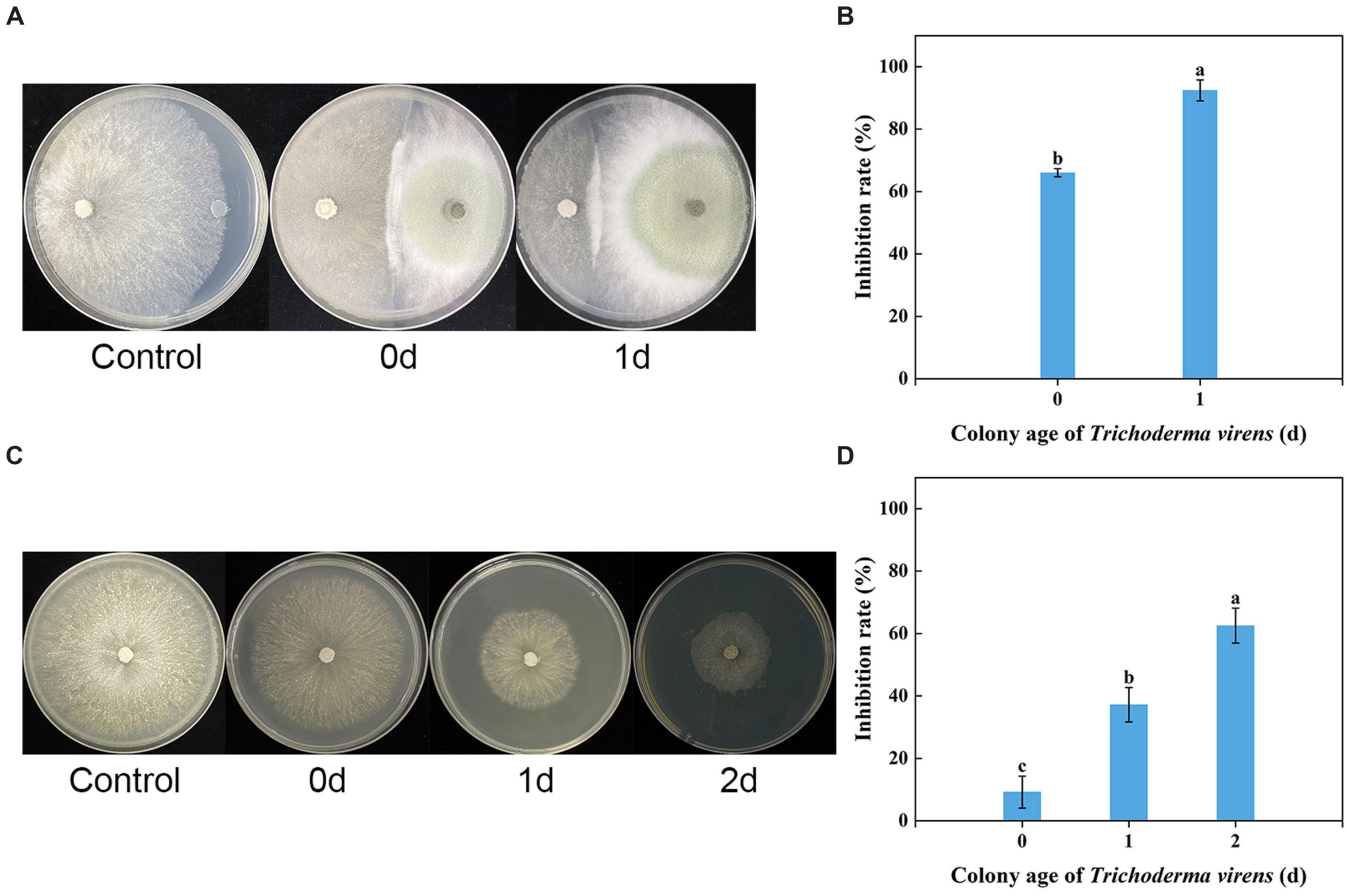
Dual culture assay and volatile organic compound antifungal bioassay of *Trichoderma virens* WJ561 against *V. pyri* G1H. **(A)** Phenotype and **(B)** inhibition rate of G1H co-cultured with 0-day-old and 1-day-old WJ561 for 3 days in dual culture assay. **(C)** Phenotype and **(D)** inhibition rate of G1H affected by VOCs of 0, 1, and 2-day-old WJ561. Error bars indicated standard errors of the means of three repeated experiments. According to Duncan’s multiple range test, columns marked by different letters represented statistically different (*p* < 0.05).

The *in vitro* dual culture assay showed that *T. virens* WJ561 was able to suppress the mycelial growth of *V. pyri* G1H on the PDA plate, with inhibitory rates of 66.02 and 92.41% on day 3 after the treatments (Figures 3A,B). The WJ561 strains formed antagonistic lines with G1H and progressively covered the growth area of G1H. The significant reductions in the colony area of the pathogen G1H occurring in the 1-day-old WJ561 colony plate suggested that the antagonistic ability depends on the colony age of *Trichoderma*.

#### 3.3.2 Antifungal bioassay of volatile organic compounds

We tested whether *T. virens* WJ561 releases inhibitory volatile organic compounds to *V. pyri* G1H (Figures 3C,D). Because *Trichoderma* grows much more slowly than pathogens G1H, we initiated *Trichoderma* culture 1 and 2 days before co-cultivation, to ensure that WJ561 produces sufficient VOCs. There were differences in the level of G1H inhibition among the different colony ages of WJ561. Notably, all treatments showed less than 65% inhibition (Figure 3D).

### 3.4 Antifungal potency of *Trichoderma virens* WJ561 cell-free supernatant

**Figure 4:**
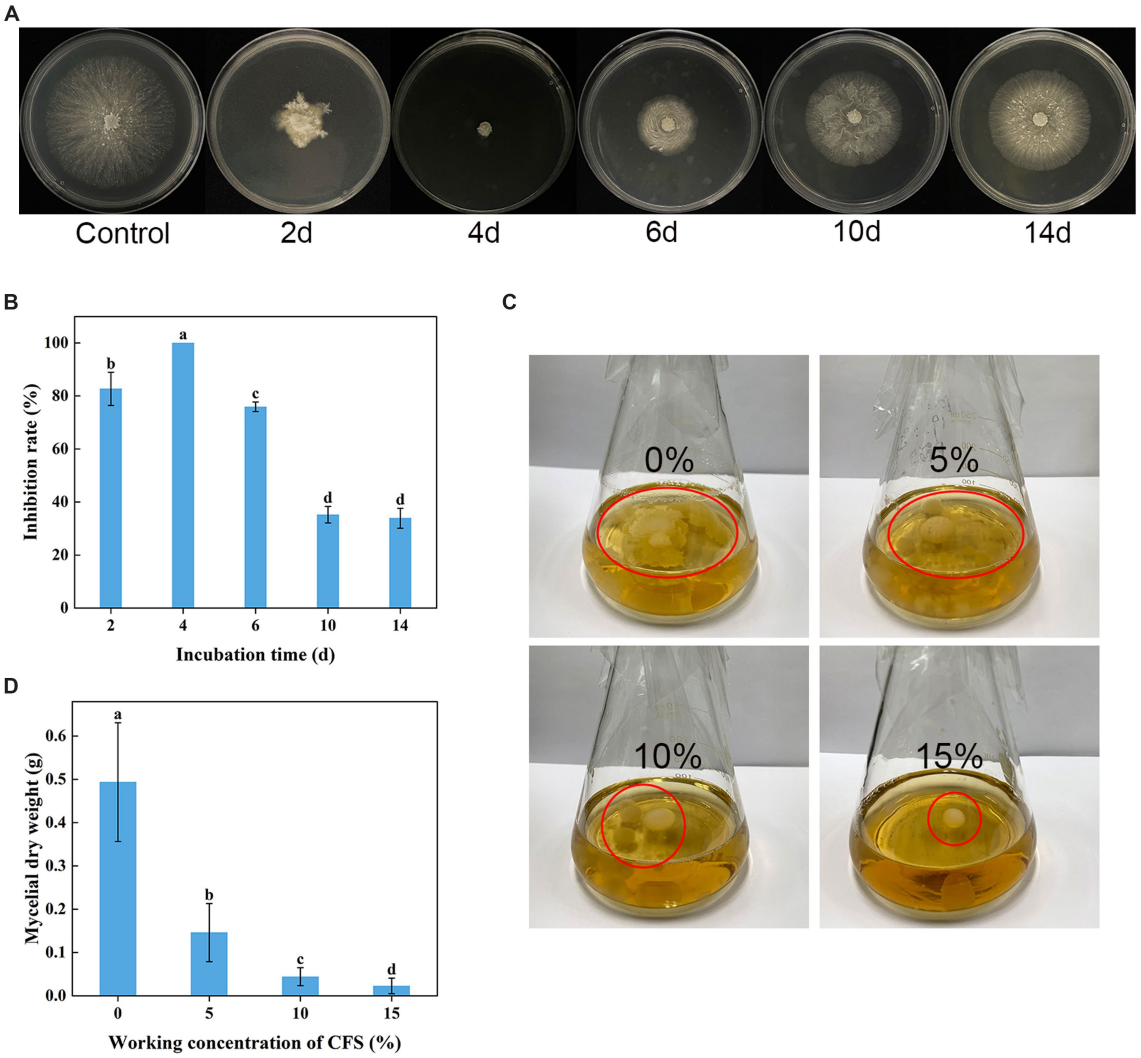
Inhibition of *Valsa pyri* G1H mycelial growth by the cell-free supernatant (CFS) of *Trichoderma virens* WJ561. **(A)** Colony size and **(B)** growth inhibition rate of G1H in the presence of CFS for different incubation times. **(C)** Size of the mycelia ball grown in the presence of different CFS concentrations. **(D)** mycelial dry weight. The data shown are meant ± standard deviations (n = 3). According to Duncan’s multiple range test, columns marked by different letters are represented as statistically different (p < 0.05).

As shown in Figures 4A,B, WJ561 CFS had a significantly inhibitory effect on the mycelial growth of *V. pyri* on PDA in Petri dishes after treatments for 48 h. However, the incubation time is a significant factor influencing the antifungal potency of WJ561 CFS. There were significant differences among WJ561 CFS prepared by different fermentation times in inhibiting mycelial growth of G1H, with the highest inhibitory rate of 100% observed in 4-day-old WJ561 CFS. The results indicated that potent antifungal activity has the potential for further research and development. In addition, we investigated the impact of varied concentrations of WJ561 CFS on the vegetative growth of G1H in a PDB liquid medium. It was observed that the mycelial ball in the CFS treatment group was smaller than the control (Figure 4C). The size of the mycelial ball in the treatment group was negatively associated with the CFS concentration. At 15% of CFS, the dry weight of mycelia was only 4.6% of that in the control group (Figure 4D).

### 3.5 Effect of *Trichoderma virens* WJ561 cell-free supernatant on controlling pear Valsa canker caused by *Valsa pyri* in detached leaves and twigs

**Figure 5:**
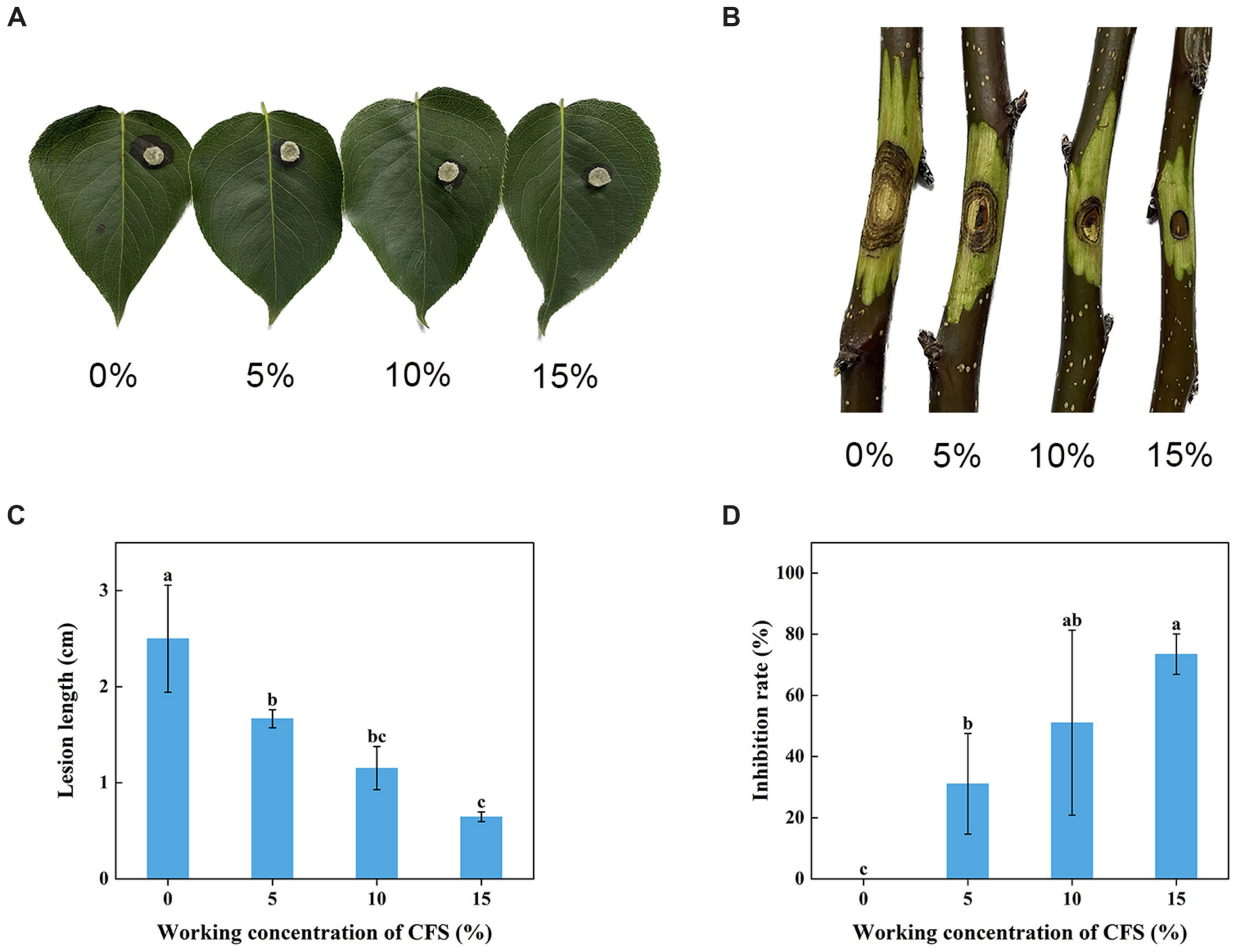
Effect of different concentrations of *Trichoderma virens* WJ561 CFS on the pathogenicity of *Valsa pyri* G1H. **(A)** Development of lesion on the detached leave infected with G1H after being treated with WJ561 CFS. **(B)** Development of lesion on the detached twigs infected with G1H after being treated with WJ561 CFS. **(C)** Canker lesion length of twigs after inoculation of a G1H plug treated with WJ561 CFS. **(D)** Disease reduction percentage rate caused by G1H on twigs treated with WJ561 CFS. The data shown are meant ± standard deviations (*n* = 3). According to Duncan’s multiple range test, columns marked by different letters are represented as statistically different (*p* < 0.05).

The WJ561 CFS was effective in preventing the expansion of pear Valsa canker lesions on leaves and twigs (Figures 5A,B). Compared to the control group treated with PBD, the lengths of lesions of CFS-treated twigs were observed to be significantly shorter on the 5th day, and there was a 73.5% decrease in lesion expansion rate after being treated with 15% CFS (Figures 5C,D). The CFS treatment was able to reduce hyphal growth in the tissues, thereby decreasing the inoculum density of the pathogen. The results indicated that WJ561 can be an efficient biocontrol agent of pear Valsa canker.

### 3.6 The stability of the *Trichoderma virens* WJ561 cell-free supernatant

**Figure 6:**
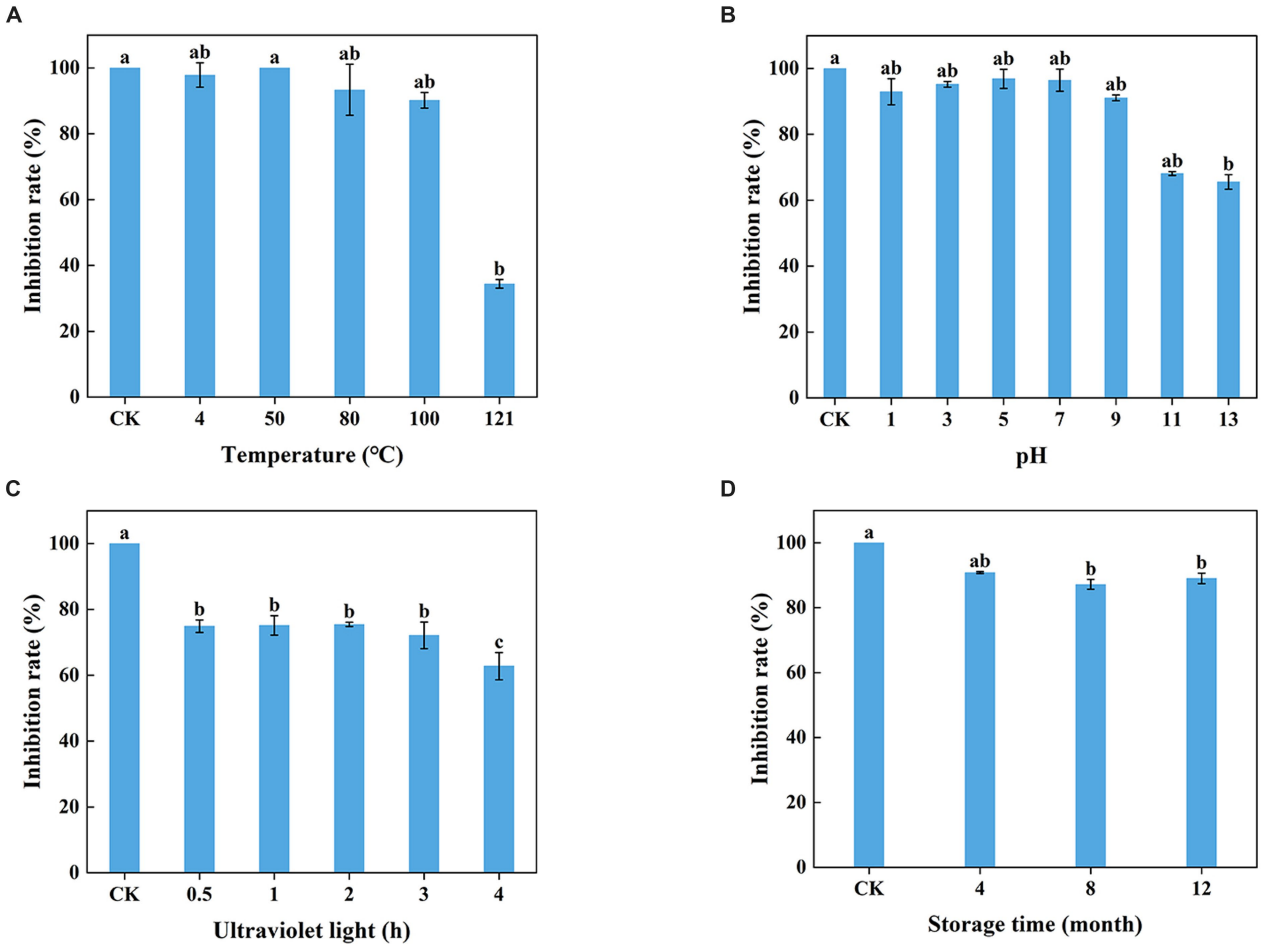
Stability of *T. virens* WJ561 cell-free supernatant. **(A)** Temperatures. Different letters above the bars indicate significant differences within the temperature treatment group according to the Kruskal–Wallis test (*H* = 13.876, *p* < 0.05). **(B)** pH values. Different letters above the bars indicate significant differences within the pH values treatment group according to the Kruskal–Wallis test (*H* = 19.479, *p* < 0.05). **(C)** Ultraviolet light treatment time. Different letters above the bars indicate significant differences within the ultraviolet light treatment group according to Duncan’s multiple range test (*p* < 0.05). **(D)** Storage time. Different letters above the bars indicate significant differences within the different storage time treatment groups according to the Kruskal–Wallis test (*H* = 9.804, *p* < 0.05). The experiments of all treatment groups were conducted three times, and the data shown are meant ± standard deviations (*n* = 3).

The WJ561 CFS appeared to be insensitive at 100°C and lower but sensitive to higher temperatures with significantly reduced activity at 121°C (Figure 6A). The lowest inhibitory rates were observed on pH 13, and ultraviolet treatment for a longer time decreased the antagonistic effect (Figures 6B,C). No significant difference was observed in the inhibitory effect of WJ561 CFS against *V. pyri* G1H when stored at 4°C within 4 months. However, the inhibition rate observed at 8 months was slightly lower than that at 12 months, which could be attributed to variations in the batches of CFS. Although the antagonistic effect decreased over time, the inhibition rate remained at approximately 89% even after a year (Figure 6D).

### 3.7 Effect of *Trichoderma virens* WJ561 cell-free supernatant on *Valsa pyri* morphology

**Figure 7:**
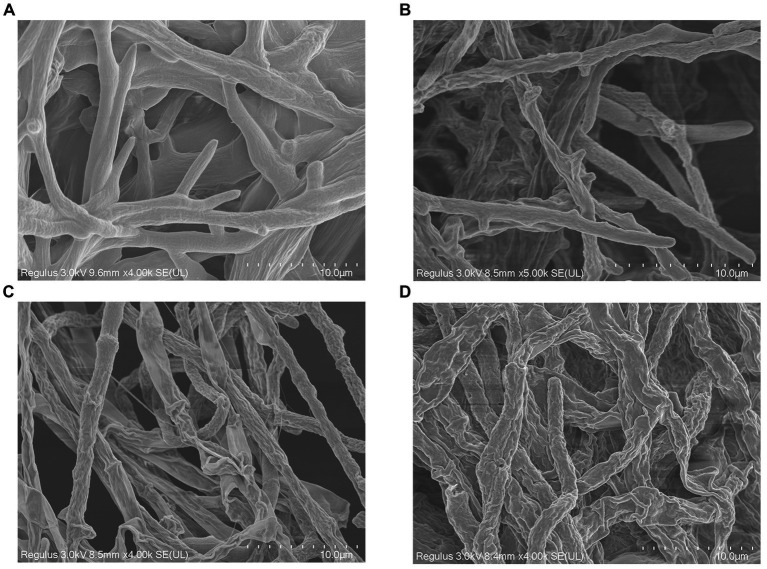
Effect of cell-free supernatant treatment on the morphology of *Valsa pyri* G1H observed by scanning electron microscopy. G1H mycelia treated with **(A)** 0% CFS, **(B)** 5% CFS, **(C)** 10% CFS, and **(D)** 15% CFS.

Scanning electron microscopy was applied to evaluate the morphological alteration to confirm whether the mycelial morphology of *V. pyri* G1H was affected by WJ561 CFS. After incubation at 25°C, a typical morphology with rougher, more sunken, and shriveled surface structure was observed in mycelia treated with CFS compared to the control group (Figure 7). Moreover, a more significant contraction and disruption could be observed when the mycelia were treated with 15% CFS, which might result in membrane permeability and loss of cytoplasmic contents (Figure 7D).

### 3.8 Determination of plasma membrane integrity, MDA content, and cellular leakage of *Valsa pyri*

**Figure 8:**
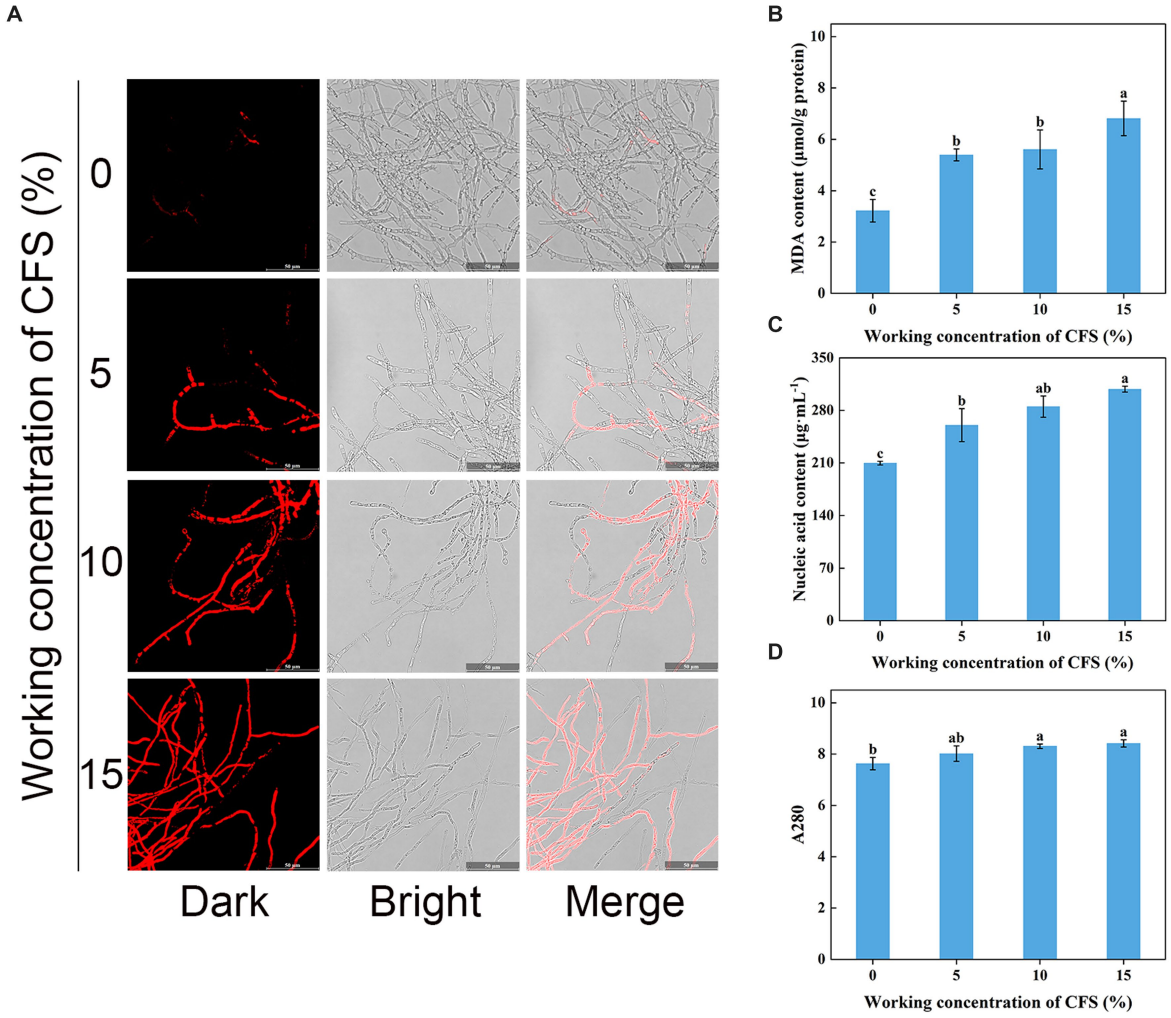
Effect of *T. virens* WJ561 cell-free supernatant (CFS) on hyphal cytomembrane integrity of *Valsa pyri* G1H. **(A)** Hyphae were stained with PI and observed with a fluorescence microscope. **(B)** MDA content. **(C)** Nucleic acid content. **(D)** Soluble protein content as measured by A280. Vertical bars represent standard deviations of the means (*n* = 3). Columns followed by different letters within MDA content and A280 are statistically different according to Duncan’s multiple range test (*p* < 0.05). Different letters above the bars indicate significant differences within nucleic acid content according to Duncan’s multiple range test (*p* < 0.05).

Shriveled hyphae observed in the WJ61 CFS treatment group showed that CFS might cause hyphal cell membrane damage and cell mortality. A DNA-staining fluorescent probe, PI, was used to determine whether cells have lost plasma membrane integrity by detecting red fluorescence. The results revealed that more hyphae exhibited red fluorescence in the treated group than in the control group (Figure 8A). This indicated that the number of hyphae losing plasma membrane integrity increased with the increase in CFS. It was observed that 10% CFS was sufficient to cause significant damage to the cell membrane of hyphae, thereby highlighting the potential of CFS in disrupting cellular membranes.

To delve into the mechanism of CFS-induced membrane damage, we measured the MDA content, which is one of the essential markers when the membrane system suffers from stress. Our findings concluded that the MDA content in the treated group was higher than that of the control group (Figure 8B), demonstrating that CFS promoted the oxidation of hyphae of *V. pyri* and generated the lipid peroxidation end product MDA. The exudation of cellular contents has been thought to respond to damage to the plasma membrane. To better investigate the oxidative effect of CFS on *V. pyri*, the nucleic acid and protein leakage of G1H was detected. The results showed that increased contents of nucleic acid and soluble protein were observed in the CFS-treated group (Figures 8C,D). Overall, these results indicated that the WJ561 CFS could disrupt the integrity of the hyphal membrane of *V. pyri* by promoting the occurrence of peroxidation damage.

### 3.9 Detection of reactive oxygen species accumulation, enzyme activity, and relative expression levels of producing genes

**Figure 9:**
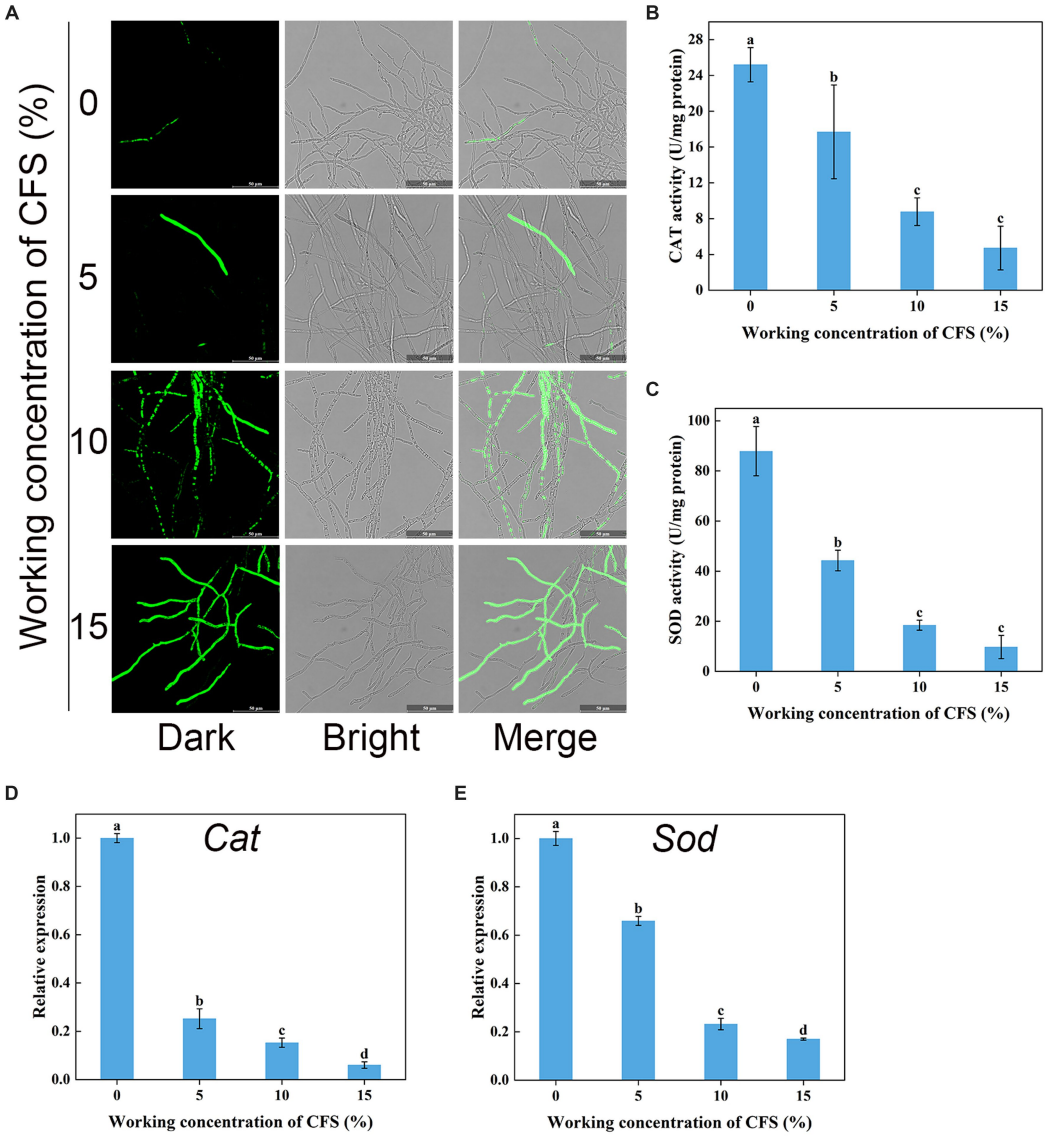
Effects of cell-free supernatant (CFS) on ROS accumulation and the enzyme activities involved in scavenging ROS in *Valsa pyri*. **(A)** The images of ROS accumulation were recorded using confocal laser microscopy. **(B)** The activity of ROS scavenging enzymes CAT in hyphal cells. **(C)** The activities of ROS scavenging enzymes SOD in hyphal cells. **(D)** Gene expression levels of *Cat*. **(E)** Gene expression levels of *Sod*. According to Duncan’s multiple range test (*p* < 0.05), different letters above the columns indicate significant differences within each group.

Hyphal cells with elevated ROS levels have been shown to generate membrane damage (Zhao et al., 2022). We next wanted to investigate whether WJ561 CFS treatment increases ROS production and oxidative stress in *V. pyri*. H_2_DCFDA, a well-known fluorescence probe, is used to detect intracellular ROS. As shown in Figure 9A, it was normal to observe that the presence of ROS in hyphal cells was negligible in the control group, while much stronger fluorescence was observed in the treated group. Moreover, the fluorescence intensity enhanced significantly with the increase in the concentration of the WJ561 CFS, indicating that CFS could strongly induce cells to produce ROS in a dose-dependent manner. In general, the accumulation of ROS in cells is usually linked with an increase in the scavenging capacity. We next assessed the enzyme activity of catalase and superoxide dismutase and the expression levels of related genes in treated hyphae. However, such a decrease in the enzyme activities of CAT and SOD was detected by CFS treatments (Figures 9B,C). Compared with the control group, the expression levels of these two related genes *Cat* and *Sod* were also significantly downregulated by qRT-PCR analysis (Figures 9D,E). These findings indicated CFS-induced ROS accumulation in membrane cells by the inhibition of ROS-scavenging pathways, leading to hyphae membrane peroxidation damage.

### 3.10 Untargeted metabolic analysis of *Trichoderma virens* WJ561

**Figure 10:**
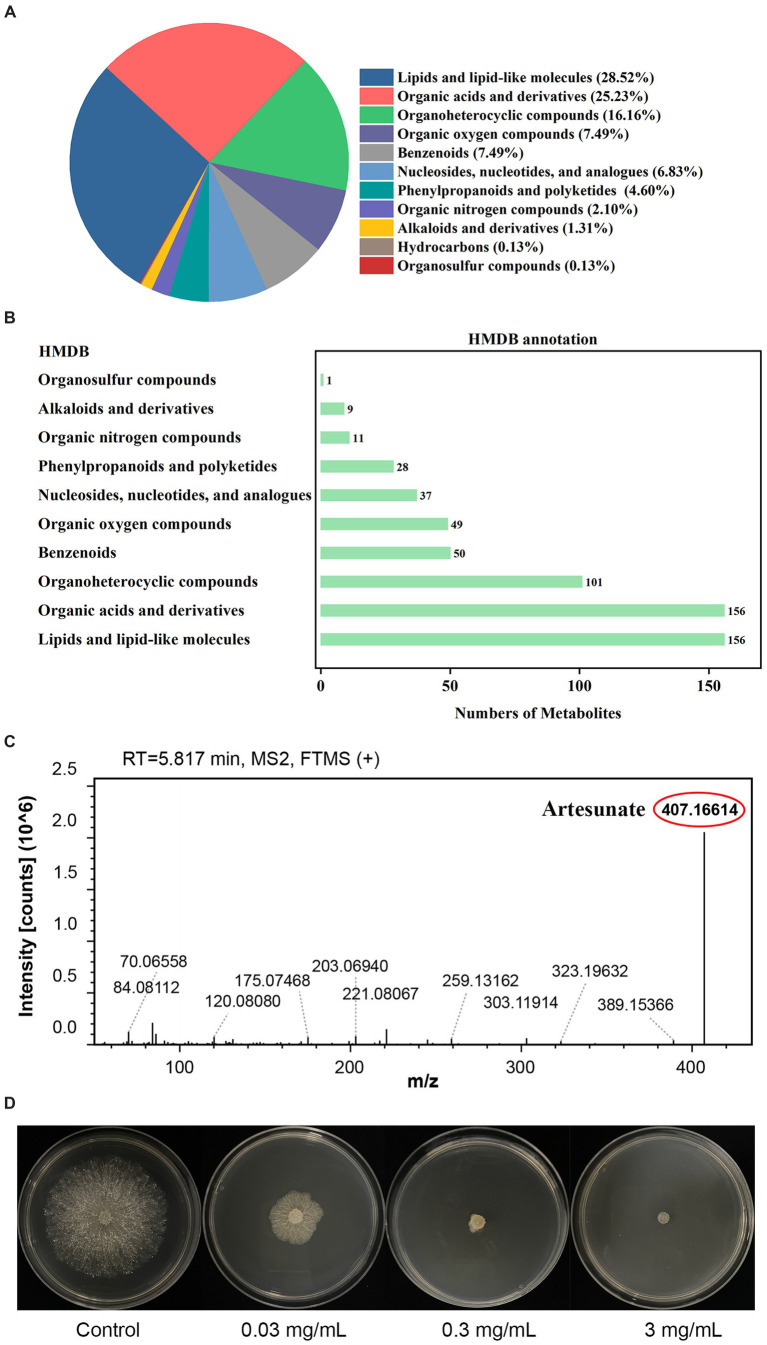
**(A)** Classification and **(B)** annotation of metabolites of *T. virens* WJ561. **(C)** Mass spectra of artesunate. **(D)** Antagonistic activity of artesunate against *V. pyri* G1H.

To investigate the metabolites through which the WJ561 CFS exerted its effects in controlling *V. pyri* G1H, we performed an untargeted metabolomic analysis of CFS by LC–MS. In total, 1,142 metabolites were identified, including 798 in the positive mode and 344 in the negative mode. The list of each metabolite in the positive and negative modes is presented in Tables S1 and S2, respectively. At the class 1 level, 761 metabolites were mainly composed of 217 lipids and lipid-like molecules, 192 organic acids and derivatives, 123 organoheterocyclic compounds, 57 organic oxygen compounds, 57 benzenoids, 52 nucleosides, nucleotides, and analogs, 35 phenylpropanoids and polyketides, 16 organic nitrogen compounds, 10 alkaloids and derivatives, 1 hydrocarbon, and 1 organosulfur compound (Figure 10A). Pathway analysis showed that these metabolites were significantly enriched in 10 HMDB pathways, including lipids and lipid-like molecules and organic acids and derivatives (Figure 10B). Based on the metabolite analysis mentioned above, some lipids and lipid-like molecules may be related to reactive oxygen species balance and pathogen inhibition (Roh et al., 2017; Wang et al., 2022). This analysis detected a [M + Na]^+^ peak at m/z 407.16614 and afforded the molecular formula C_19_H_28_O_8_ (Figure 10C). We conducted a preliminary investigation into the efficacy of metabolites on *V. pyri* G1H. In the present study, the antifungal test of artesunate at 0.3 mg/mL significantly inhibited the growth of *V. pyri* (Figure 10D). In addition, 3 mg/mL lauric acid, arachidonic acid, and linoleic acid also slightly inhibited pathogenic fungi (Supplemenatry Figure S1).

## 4 Discussion

The present study examined if *T. virens* effectively inhibited *V. pyri*. Pear Valsa canker, caused by *V. pyri*. significantly affects the fruit yield, quality, and longevity of trees (Song et al., 2020; Naqvi et al., 2022). The treatment of pear Valsa canker heavily depends on the chemical fungicides. However, this may be suboptimal in fungicides because of the escalating resistance and environmental implications. Recent advancements in microorganisms as green alternatives for controlling plant pathogens have facilitated the successful development and commercialization of specific strains of *Trichoderma* for application in agriculture and industry (Busato et al., 2020; Porteous-Álvarez et al., 2023). *Trichoderma* and its metabolites are promising interventions due to their antimicrobial effects against pathogenic cells. This study investigated the antifungal activity of CFS produced by *T. virens* against *V. pyri* and the mechanism of WJ561 CFS-induced *V. pyri* hyphal membrane damage.

In this study, G1H isolated from the bark of pear was found to be closely related to *V*. *pyri* isolate GSZYpm160 (Figure 1). This *Trichoderma* isolate WJ561 showed high similarity with *T. virens* isolate YZB-1 and *T. virens* isolate TB2-7 as a potential bioagent (Figure 2). We examined the antagonistic activity of *Trichoderma* against *V. pyri* using dual culture assays, volatile antifungal bioassays, and cell-free supernatant tests (Figures 3, 4). The data showed that the most significant antibiosis of CFS of *T. virens* was observed in the 4-day-old CFS, ultimately inhibiting the mycelial growth of *V. pyri* within 2 days (Figures 4A,B). The optimized microbial incubation time, which guarantees the highest antifungal effect, was achieved. These differences in incubation time contributed to the different inhibition rates observed among *T. virens*, suggesting that the time to obtain confronting fungal colonies and differences in metabolites may account for the action mode of *T. virens* WJ561 against *V. pyri* G1H (Bertrand et al., 2014; Triastuti et al., 2021). For instance, when co-culturing strains belonging to *Cophinforma mamane* and *Fusarium solani*, changes in metabolite composition over time have been observed (Triastuti et al., 2021). Lee et al. believed that the age of the fungal culture was crucial for microbial volatile-mediated growth promotion and/or inhibition (Lee et al., 2015). We compared the inhibition of the different ages of *T. virens* WJ561 culture against *V. pyri* G1H, which could assist in the explanation of fungal interaction. In addition, a shorter fermentation period with a better antifungal effect would be cost-effective and time-efficient for the commercial production process.

In previous studies, antagonistic interactions between *Trichoderma* species and pathogens, as well as among strains of the same *Trichoderma* species, have been documented (Zhang et al., 2016; Wonglom et al., 2019). These interactions typically encompass competition for space and nutrients, secretion of inhibitory compounds, and the induction of systemic resistance. Antibiosis in *Trichoderma* was correlated with its metabolites (Keswani et al., 2013). In certain instances, the protective efficacy of *Trichoderma* against pathogenic fungi can be attributed, at least in part, to its direct antagonistic action via the secretion of antimicrobial metabolites such as chitinase and glucanase (Zhang et al., 2016; Wu et al., 2017). Some events in the *Trichoderma*–pathogen interaction may be linked to the generation of ROS. The proteinaceous elicitor sm1, secreted by *Trichoderma*, can upregulate these enzymes (chitinase and glucanase), thereby triggering ROS production (Djonovic et al., 2006). In addition, some strains of *Trichoderma virens* could produce gliotoxin to induce cell apoptosis (Zaid et al., 2022). These results suggested that the *T. virens* WJ561 may secrete similar components in the role of biological control. Therefore, further experiments were conducted to explore the potential functions and mechanisms of *T. virens* CFS against *V. pyri*.

Treatment with WJ561 CFS has been shown to inhibit the hyphal growth of *V. pyri in vitro* and impair the pathogenicity of *V. pyri* on the tested leaves and twigs (Figures 4,5). The CFS produced by WJ561 was sensitive to 121°C and ultraviolet irradiation, had the worst efficacy at pH 11 and 13, and was relatively resistant to storage time (Figure 6). Further analysis demonstrated that the inhibition of *V. pyri* growth corresponded with morphological changes in hyphae, which were shriveled and deformed by WJ561 CFS (Figure 7). Hyphal damage is a crucial fatal factor in many pathogenic fungi. ROS is the most essential and prominent inducer, which causes oxidative damage, lipid peroxidation, membrane destruction, and cell death. We observed that the increased fluorescence signal of ROS in hyphae was concentration-dependent, indicating that peroxidation was exacerbated (Figure 9A). Furthermore, the disruption of cell membranes and the alteration of cell permeability by CFS-induced ROS damage were supported by PI, MDA, and intracellular content leakage measurements (Figure 8). ROS-induced cell death is beneficial for pathogen inhibition (Cui et al., 2021; Wang and Zeng, 2022). In other words, inhibiting ROS to prevent oxidative damage may be advantageous for pathogenic fungi. Thus, this pathogen-beneficial ROS suppression effect may be driven by the pathogens themselves to scavenge ROS. It was supported by Boukaew et al., who reported that *Aspergillus parasiticus* and *A. flavus* increased tolerance to biotic stress through enhanced activities of antioxidant enzymes activities, such as POD, SOD, and CAT (Boukaew et al., 2023).

However, it remains unclear how WJ561 CFS balances its ROS-mediated responses to inhibit *V. pyri* during infection. Previous studies have shown that the non-volatile metabolites of *T. virens* ZT05 effectively downregulated the expression of antioxidant proteins in *Rhizoctonia solani* mycelia after 24 h, thereby inhibiting the growth of *Rhizoctonia solani* (Halifu et al., 2020). Similarly, the symbiotic interaction between *T. virens* and maize resulted in a decrease in secreted peroxidase, which indicated that *T. virens* may restructure plant secretome responses as a strategy to suppress plant immunity (Nogueira-Lopez et al., 2018). Therefore, it is speculated that the interaction between *T. virens* and *V. pyri* may be the inhibiting effect of their antioxidant processes. Then, the biological function of the antioxidant enzymatic system was explored. Our results demonstrated that the activities of CAT and SOD in *V. pyri* of WJ561 CFS began to decrease in a dose-dependent manner (Figures 9B,C), with significantly downregulated expressions of CAT and SOD-related genes compared with the control group (Figures 9D,E). Overall, these results demonstrated that the impaired activation of immune responses in *V. pyri* might facilitate a heightened ROS accumulation, culminating in more extensive and severe oxidative damage progression.

A significant number of microorganisms have demonstrated promising results in the prevention and treatment of disease, acting through the production of biologically active metabolites. The effect of metabolites on the antagonism ability of microorganisms against fungal pathogens is well documented. Three metabolites, namely, iberverin, hexanoic acid, and 2-methylvaleraldehyde produced by *Bacillus atrophaeus* strain HF1, have robust antifungal activity against *V*. *pyri* (Yuan et al., 2023). VOCs, 2,4-di-tert-butylphenol and 4-methyl-1-pentanol, released by *Aspergillus niger* strain La2, are promising novel candidate biocontrol agents for the control of pear Valsa canker (Shi et al., 2024). *T. virens* and its emitted metabolites, including linolenic acid, fumaric acid, and citric acid, were reported to possess antifungal activity and influence pathogen metabolism (Gan et al., 2022). The metabolites are effective antifungal compounds that interfere with critical processes in target pathogens and enhance the competitive ability of biocontrol strains. The usage of WJ561 CFS can be effective in controlling *V. pyri*. Due to the diversity and complexity of the categories, further research is required to determine which metabolites exert an effect. We performed metabolomic assays on the WJ561 CFS and identified 1,142 metabolites in positive and negative ionization mass spectrometry. Lipids and lipid-like molecules were the most abundant in CFS (Figure 10A). Some of these natural lipids and lipid-like molecules, such as lauric acid, arachidonic acid, and linoleic acid, have been reported to have good antibiosis properties. For instance, lauric acid has been observed to induce apoptosis in the pathogens of rice sheath blight by its impact on the fungal fatty acid metabolism, disrupting the dynamic equilibrium of ROS (Wang et al., 2022). Further studies have found that polyunsaturated fatty acids, such as arachidonic acid and linoleic acid, can aggravate lipid peroxidation and promote cell death (Huang et al., 2023; Ma et al., 2023). Moreover, the strong antifungal activities of the fermentation of *T*. *longibrachiatum* T6 on the apple *Valsa* canker pathogen *V*. *mail* may account for the role of the antagonistic lipids, such as (Z)-octadec-9-enoic acid and (Z)-13-Docosenamide (Zhang et al., 2018). A natural terpenoid product, citral, was also reported to have the capability against *V*. *mail* (Li et al., 2015). In addition, artesunate could induce cell death by ROS production (Roh et al., 2017; Barman et al., 2023). We performed targeted assays for artesunate, lauric acid, arachidonic acid, and linoleic acid and found that artesunate had the most significant inhibitory effect on *V. pyri* (Figure 10D). Although more research is required to elucidate how CFS, especially artesunate, affect *V. pyri*, our study provides a theoretical basis for the potential applications of controlling pear Valsa canker.

## Data availability statement

The datasets presented in this study can be found in online repositories. The names of the repository/repositories and accession number(s) can be found in the article/Supplementary material.

## Author contributions

YZ: Conceptualization, Data curation, Formal analysis, Investigation, Methodology, Supervision, Validation, Visualization, Writing – original draft, Writing – review & editing. YL: Methodology, Supervision, Visualization, Writing – review & editing. ZJ: Methodology, Supervision, Validation, Writing – review & editing. BL: Methodology, Visualization, Writing – review & editing. LW: Conceptualization, Data curation, Formal analysis, Methodology, Project administration, Supervision, Validation, Writing – review & editing. YH: Conceptualization, Data curation, Formal analysis, Funding acquisition, Investigation, Methodology, Project administration, Supervision, Validation, Writing – review & editing.

## Footnotes

^1^ http://www.ncbi.nlm.nih.gov/BLAST

^2^ https://www.mzcloud.org/
