# Supplementary figures and images for: Antifungal mechanism of cell-free supernatant produced by Trichoderma virens and its efficacy for the control of pear Valsa canker
Source: Front Microbiol. 2024 Apr 17;15:1377683. doi: 10.3389/fmicb.2024.1377683 (PMC11061385; doi:10.3389/fmicb.2024.1377683)

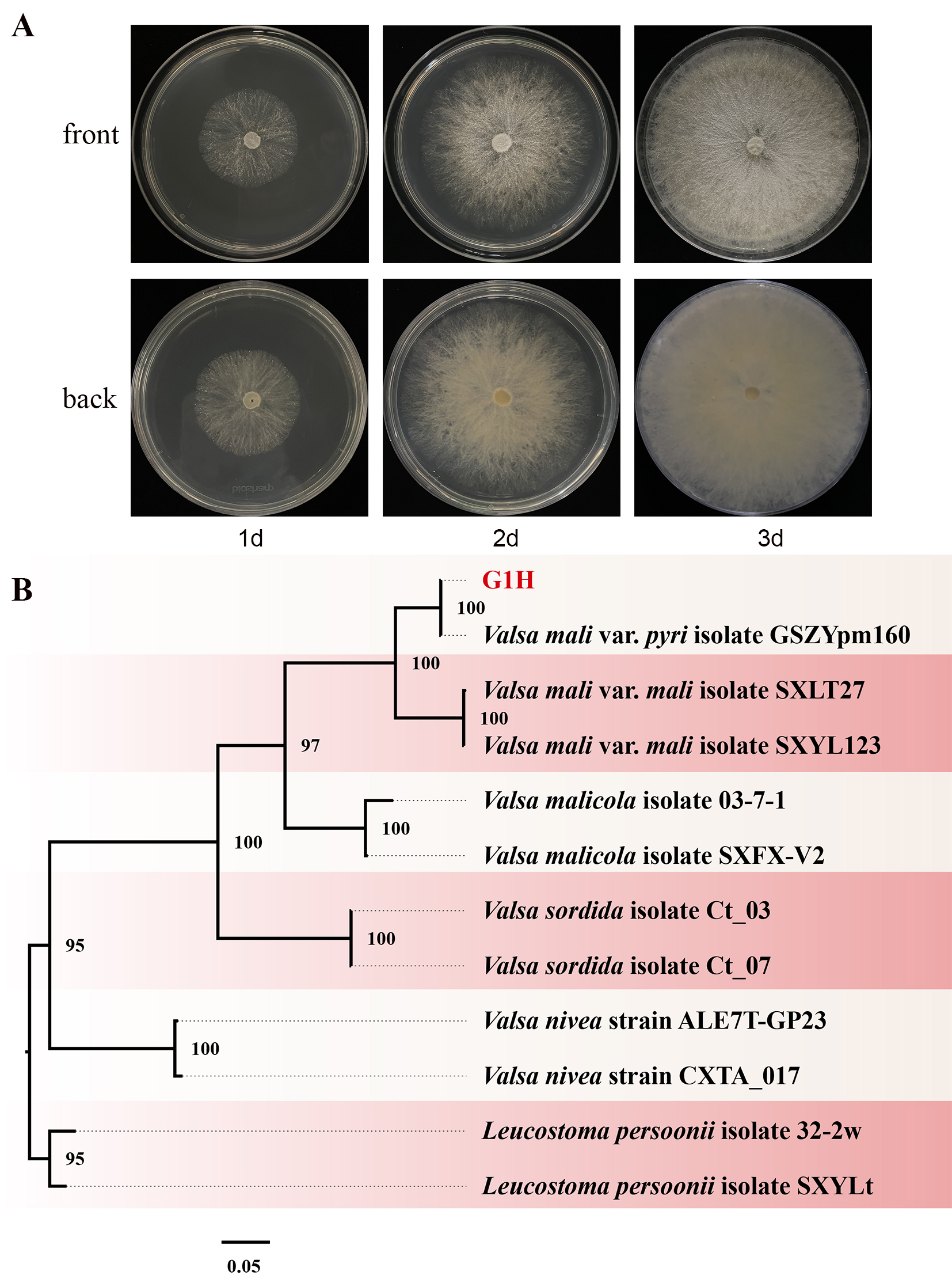

Supplement: Supplementary file 3 [file Data_Sheet_1.ZIP › Supplementary Material Presentation/Figure 1.jpg]

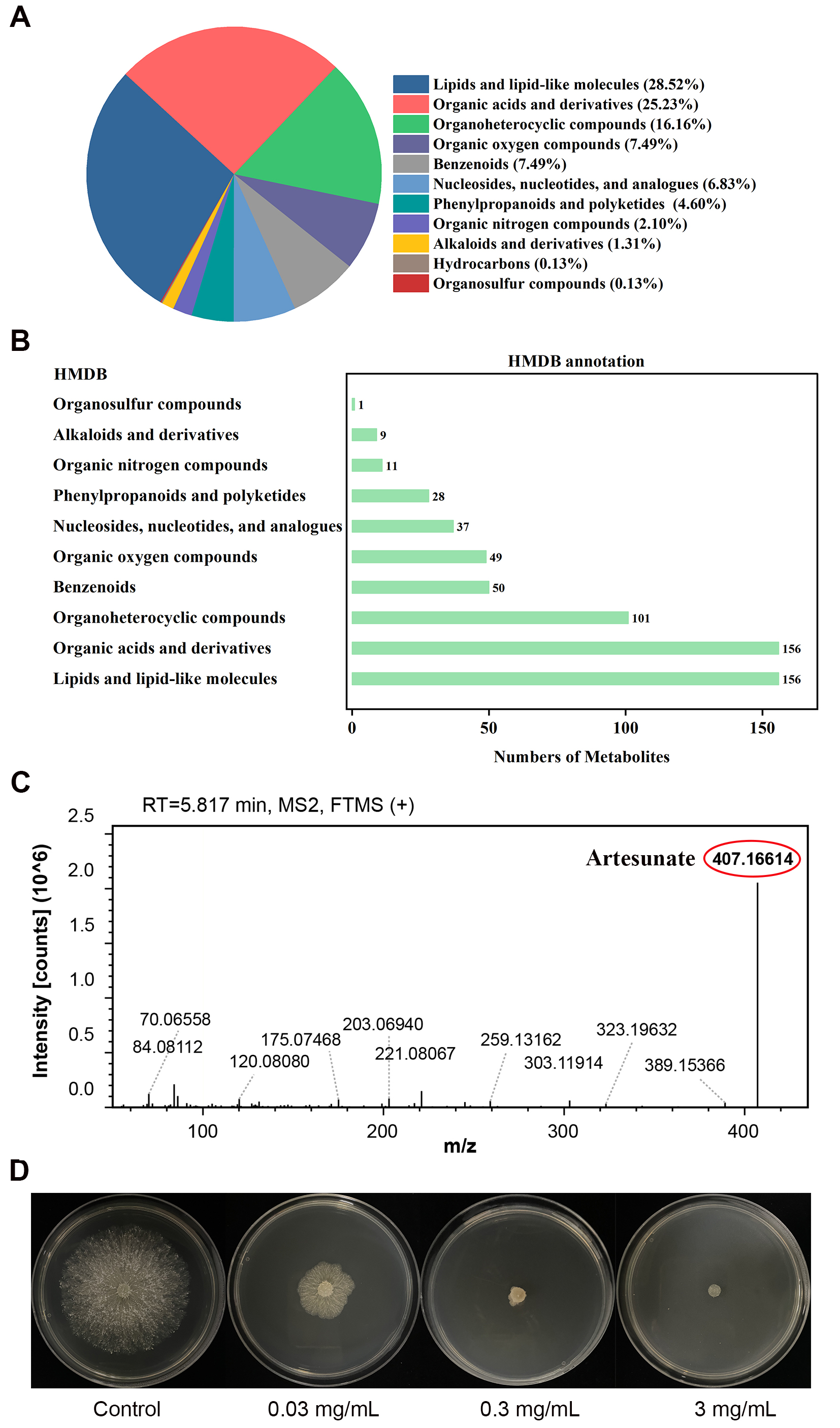

Supplement: Supplementary file 3 [file Data_Sheet_1.ZIP › Supplementary Material Presentation/Figure 10.jpg]

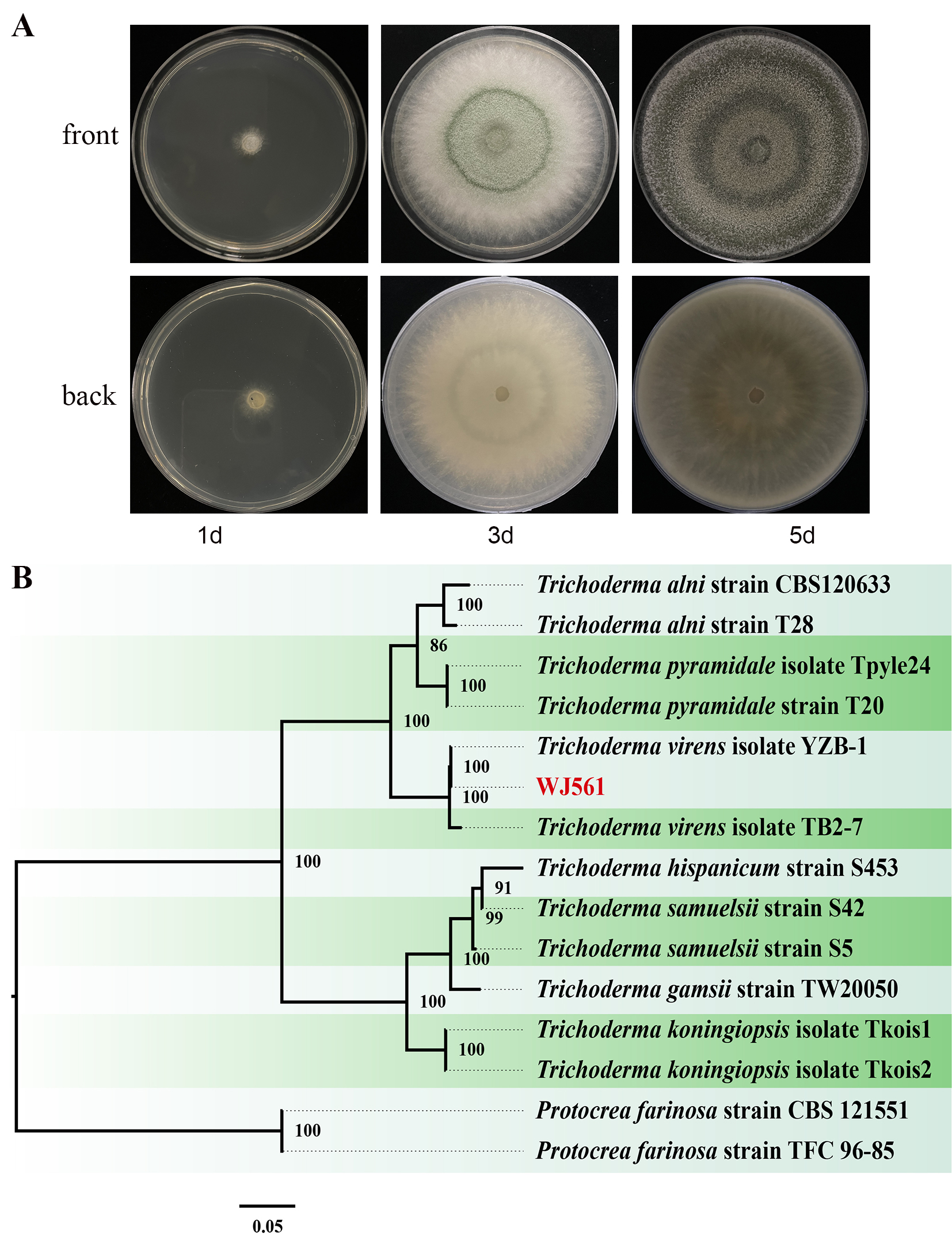

Supplement: Supplementary file 3 [file Data_Sheet_1.ZIP › Supplementary Material Presentation/Figure 2.jpg]

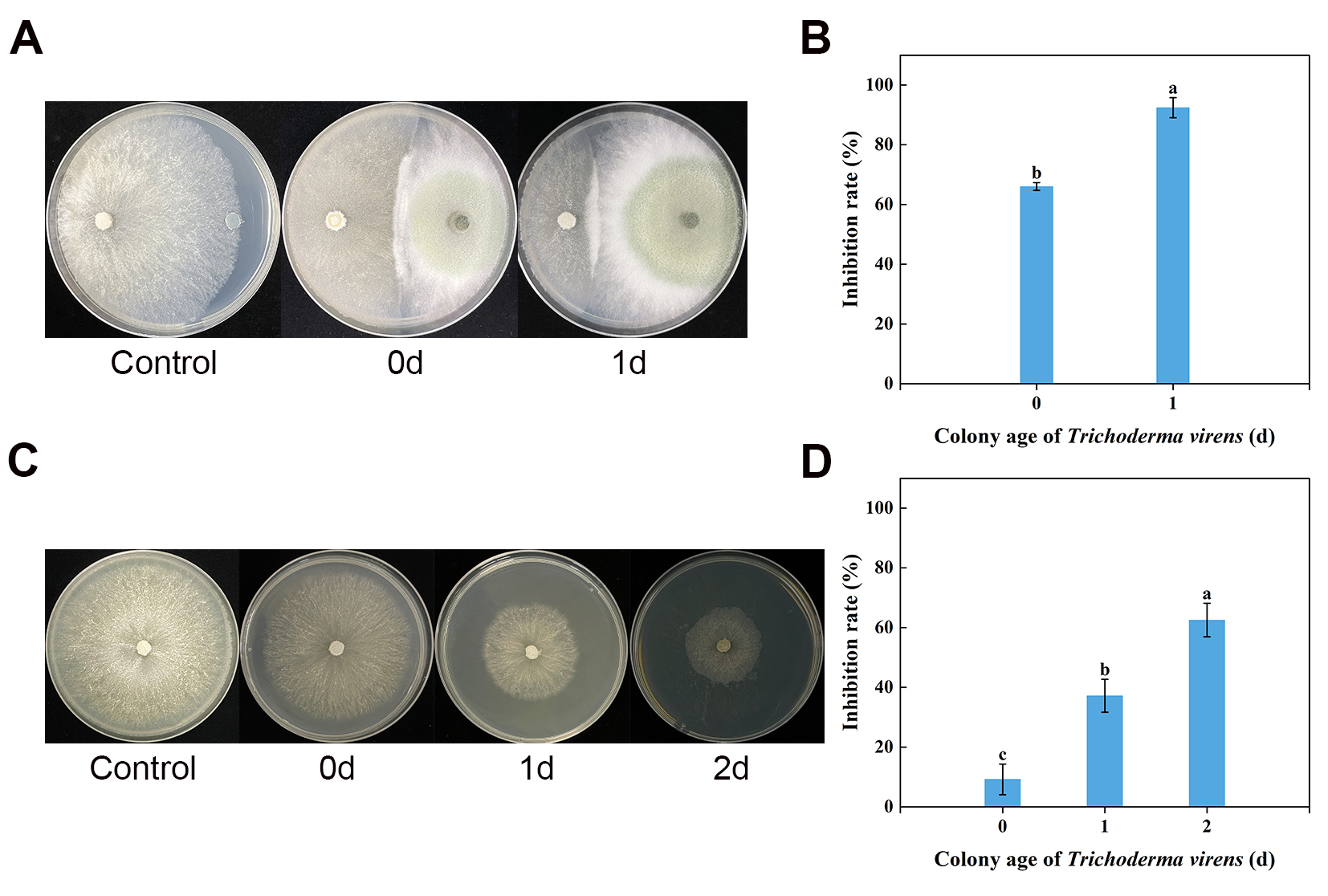

Supplement: Supplementary file 3 [file Data_Sheet_1.ZIP › Supplementary Material Presentation/Figure 3.jpg]

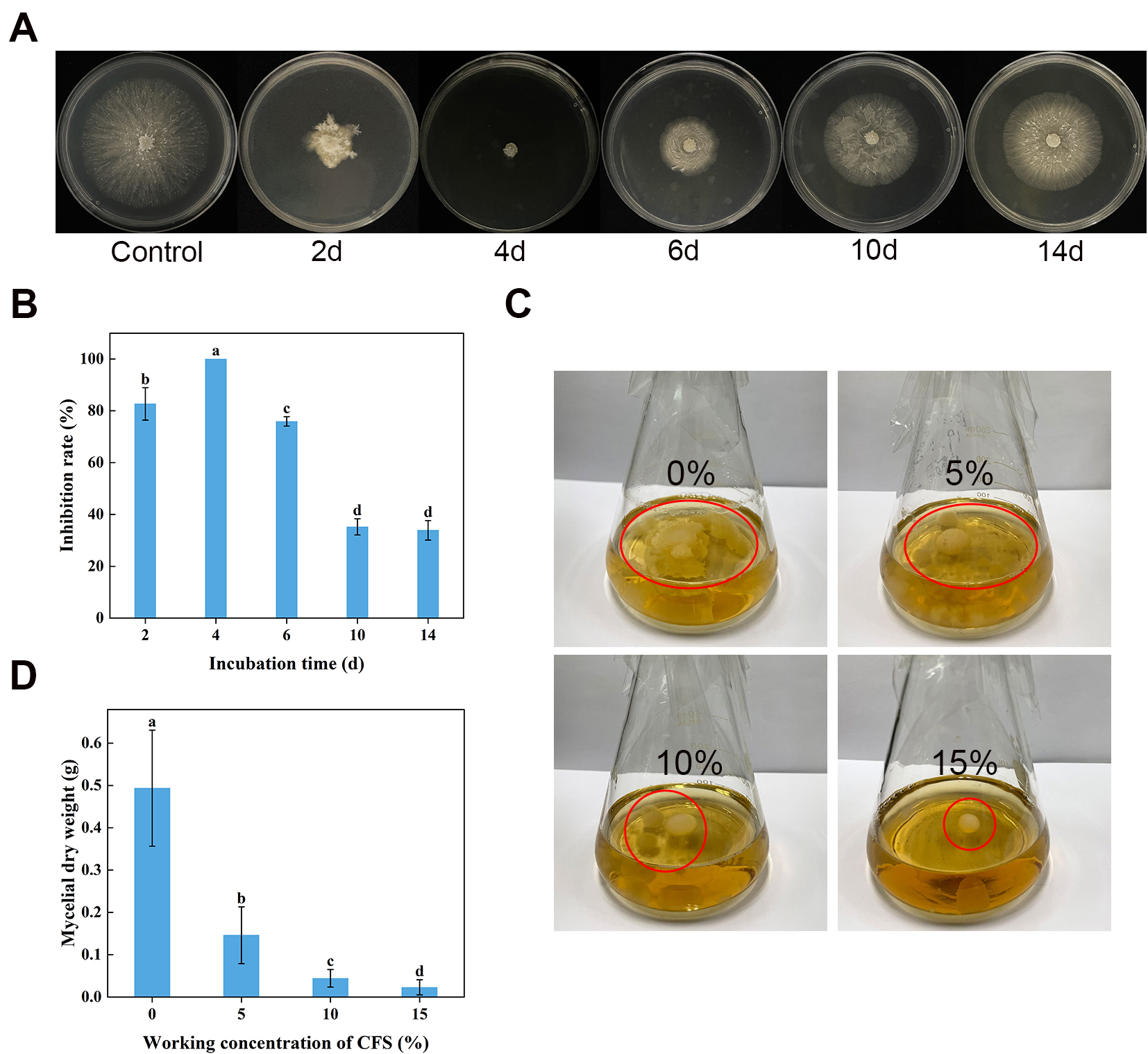

Supplement: Supplementary file 3 [file Data_Sheet_1.ZIP › Supplementary Material Presentation/Figure 4.jpg]

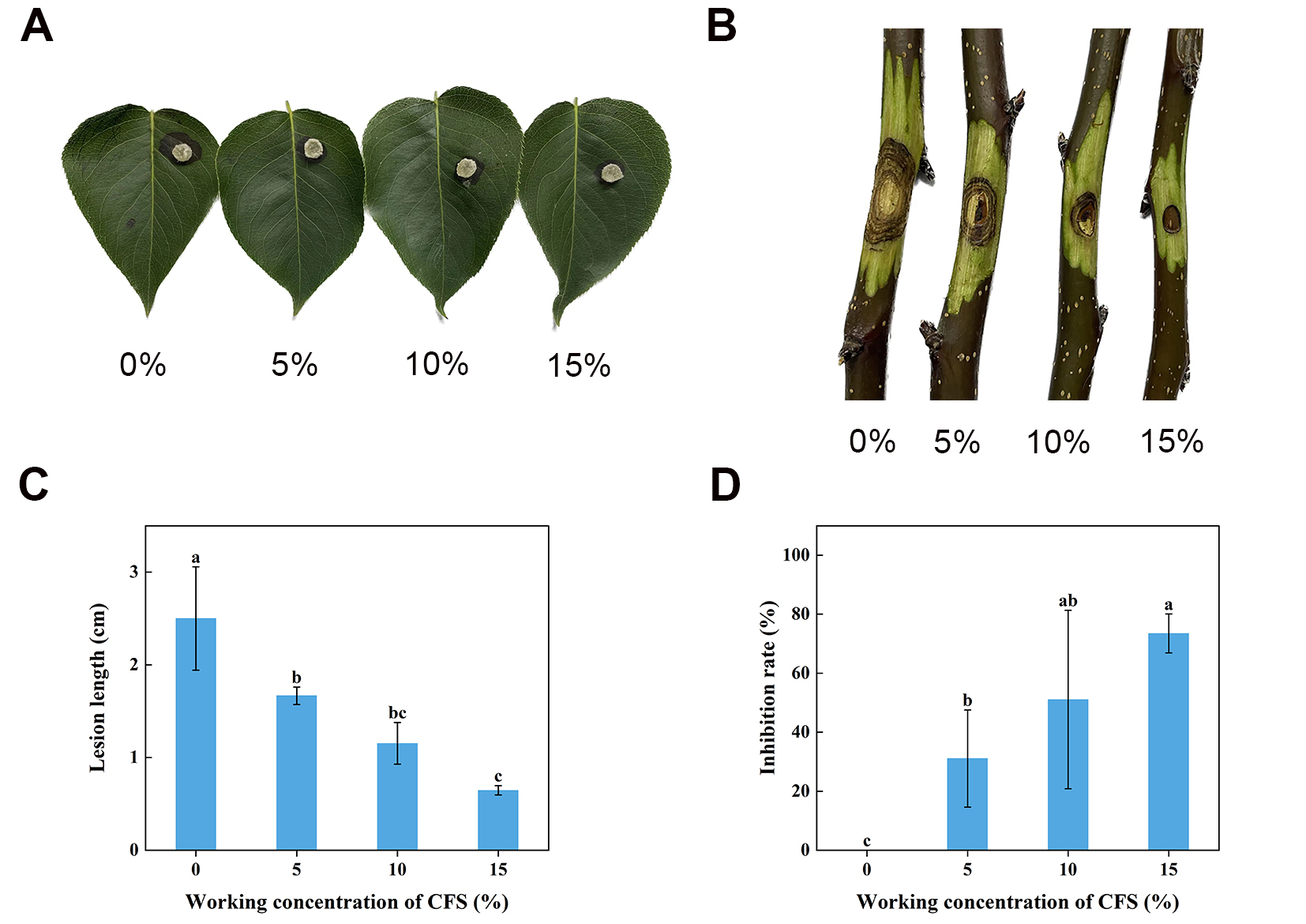

Supplement: Supplementary file 3 [file Data_Sheet_1.ZIP › Supplementary Material Presentation/Figure 5.jpg]

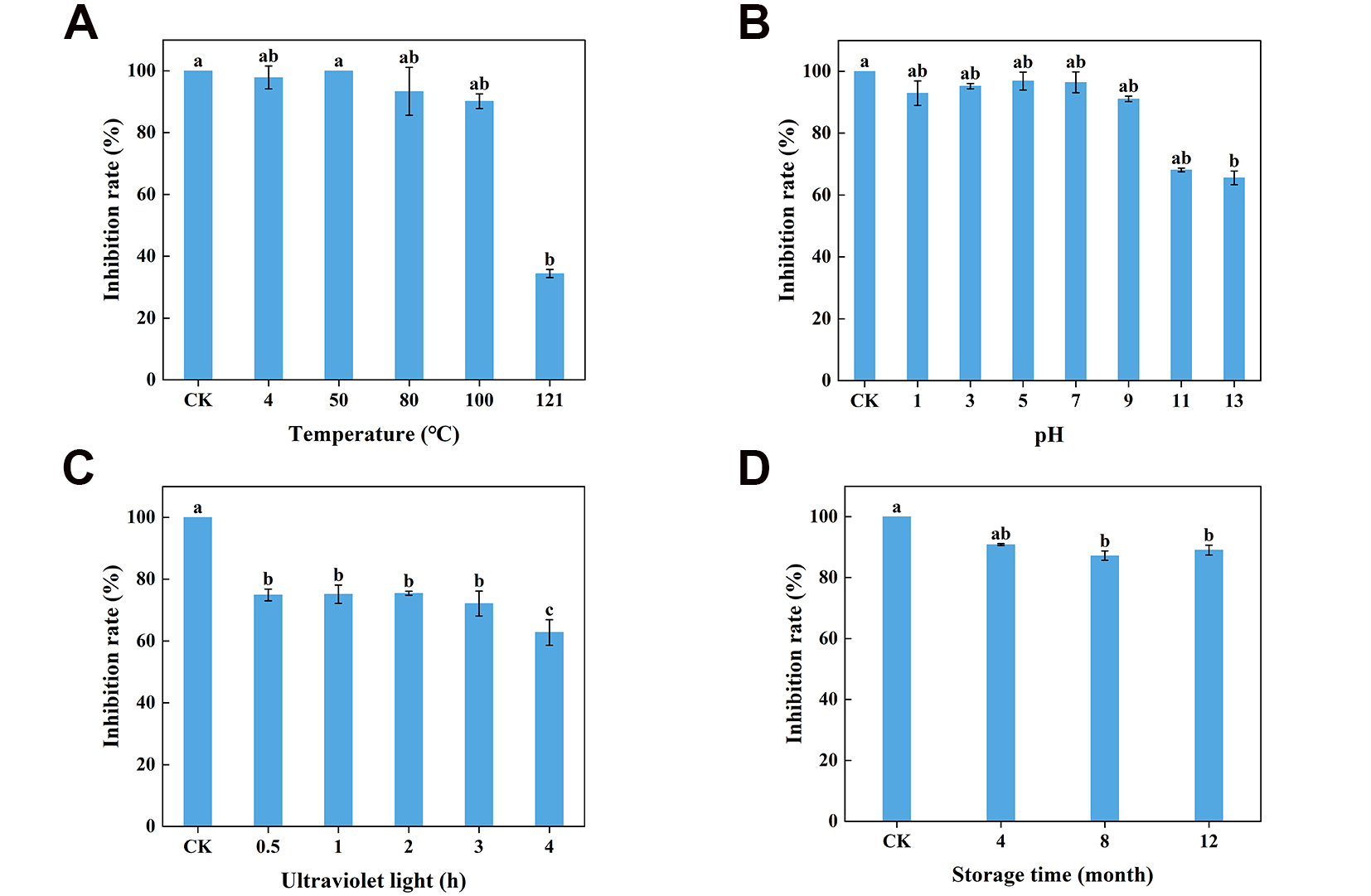

Supplement: Supplementary file 3 [file Data_Sheet_1.ZIP › Supplementary Material Presentation/Figure 6.jpg]

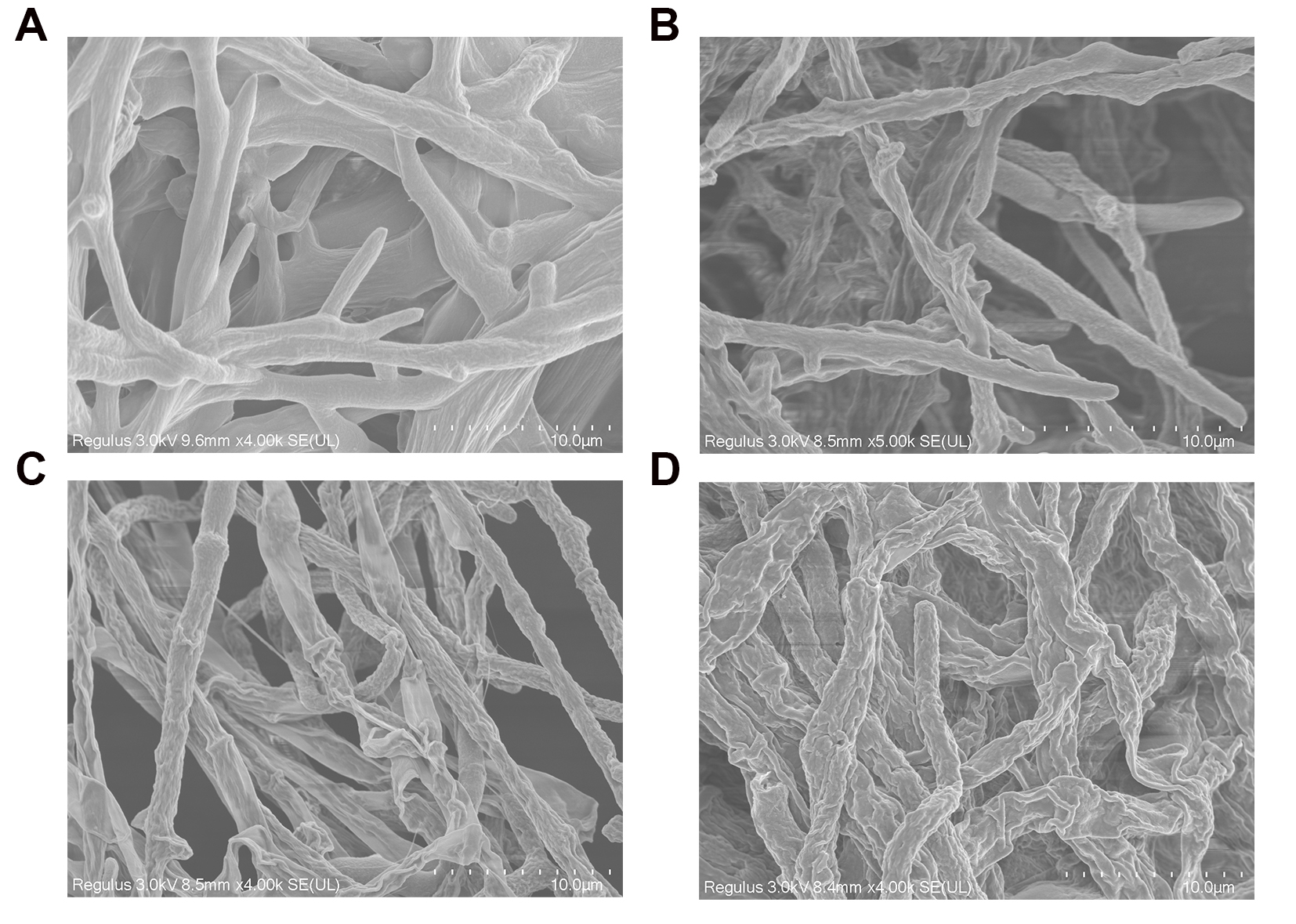

Supplement: Supplementary file 3 [file Data_Sheet_1.ZIP › Supplementary Material Presentation/Figure 7.jpg]

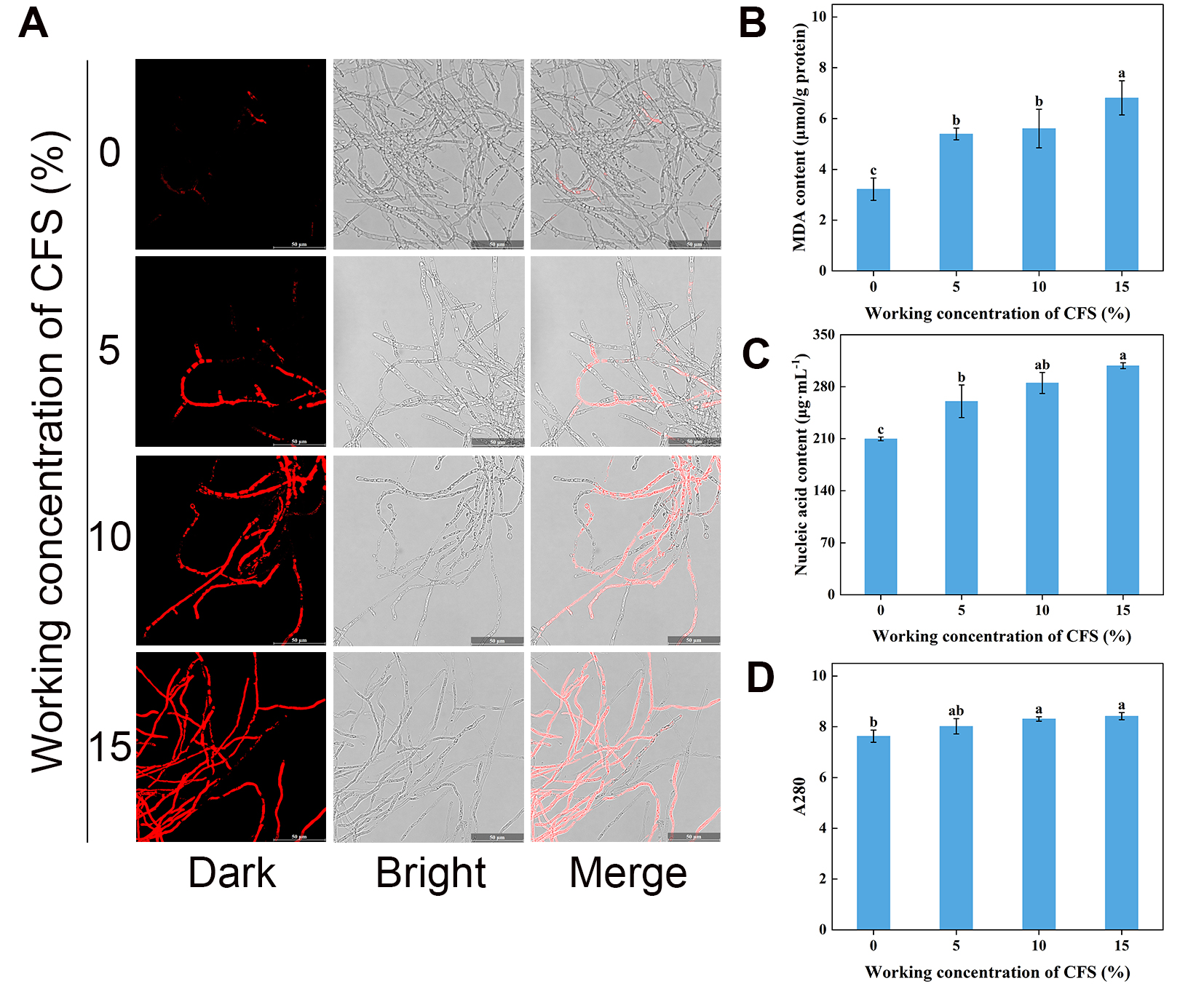

Supplement: Supplementary file 3 [file Data_Sheet_1.ZIP › Supplementary Material Presentation/Figure 8.jpg]

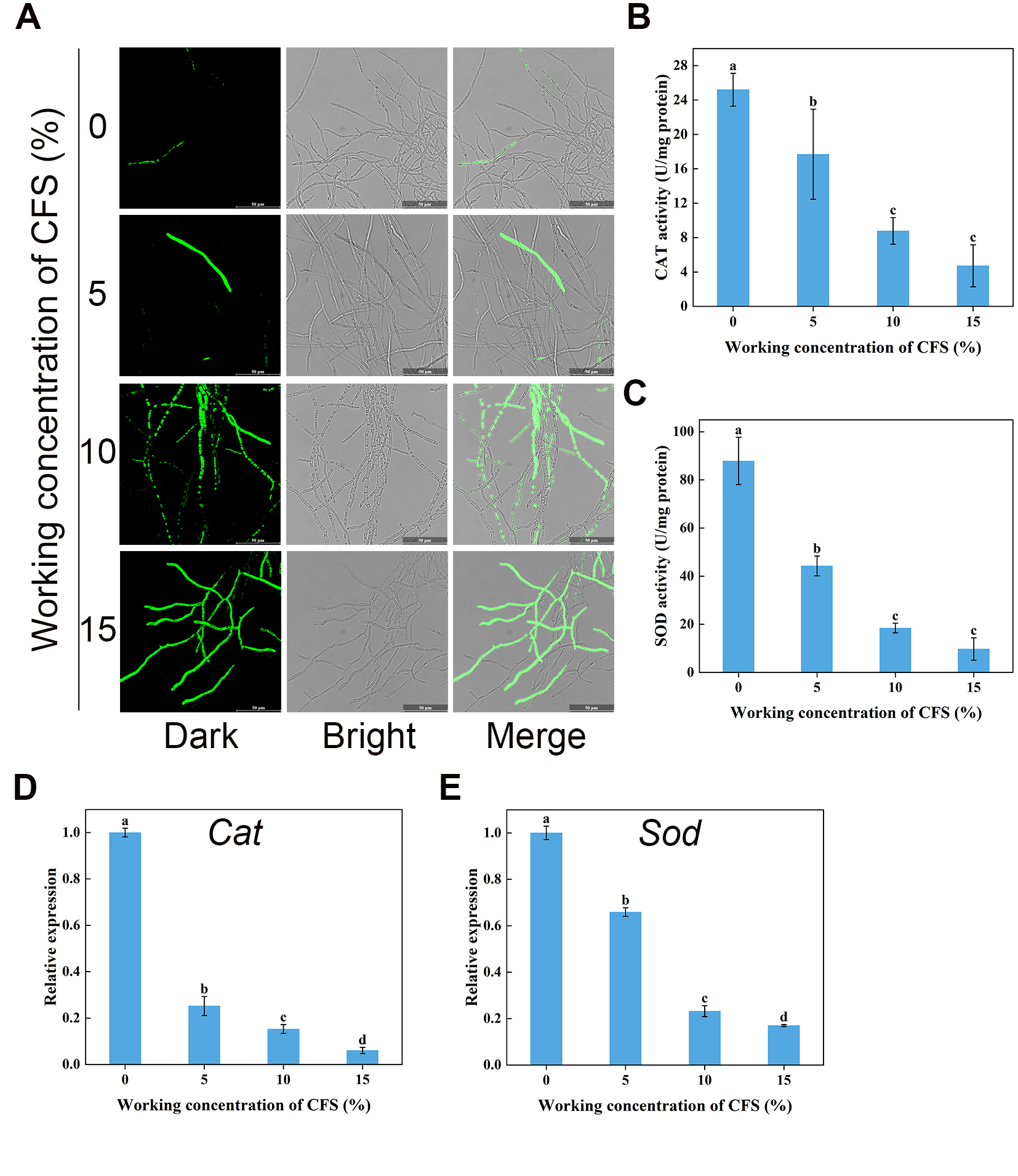

Supplement: Supplementary file 3 [file Data_Sheet_1.ZIP › Supplementary Material Presentation/Figure 9.jpg]
