# Supplementary material for: Antifungal mechanism of cell-free supernatant produced by Trichoderma virens and its efficacy for the control of pear Valsa canker
Source: Front Microbiol. 2024 Apr 17;15:1377683. doi: 10.3389/fmicb.2024.1377683 (PMC11061385; doi:10.3389/fmicb.2024.1377683)
Supplement: Supplementary file 4 [file Data_Sheet_2.DOCX]

Supplementary Material

**Antifungal mechanism of cell-free supernatant produced by *Trichoderma virens* and its efficacy for the control of pear *Valsa* canker**

Yang Zhang*, Ying Lu, Zhaoyang Jin, Bo Li, Li Wu, Yujian He

*** Correspondence:** Corresponding Author: [heyujian@ucas.ac.cn](mailto:heyujian@ucas.ac.cn), [wuli@ucas.ac.cn](mailto:wuli@ucas.ac.cn)

# Supplementary Figures

## Supplementary Figures


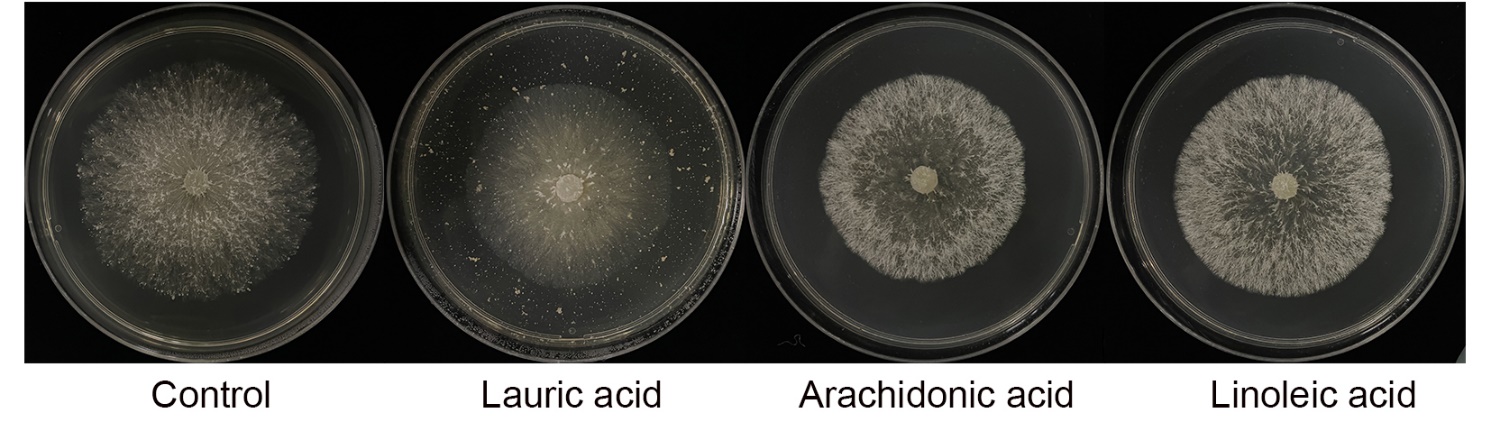


**Supplementary Figure 1.** Antagonistic activity of 3 mg/mL lauric acid, arachidonic acid, and linoleic acid against *V.* *pyri* G1H.
